# Genetically Expanded Fluorescent ^SSB^T7 RNA Polymerase in Action: Intriguing Photophysics with Enhanced RNA Production

**DOI:** 10.1021/acsomega.6c07950

**Published:** 2026-10-15

**Authors:** Subhendu Sekhar Bag, Aniket Banerjee, Sinchini Barman

**Affiliations:** † Chemical Biology/Genomics Laboratory, Department of Chemistry, 28678 Indian Institute of Technology Guwahati, Guwahati 781039, India; ‡ School of Interdisciplinary Studies and Sustainability, Indian Institute of Technology Guwahati, Guwahati 781039, India; § JBM School of Health Science and Technology, Indian Institute of Technology Guwahati, Guwahati 781039, India

## Abstract

T7 RNA polymerase (T7-RNAP) is a highly efficient transcriptional enzyme. However, it is prone to mutation at the functionally important Y571 residue, which strongly influences its functional properties. Here, we demonstrate a strategy to modulate the T7-RNAP enzyme by replacing the Y571 residue with a fluorescent triazolyl unnatural amino acid (FTUAA), ^**TCNB**^ **Ala**_**Ac**_, via genetic code expansion (GCE) technology using two orthogonal aminoacyl-tRNA synthetase/tRNA pairs. Thus, ^**TCNB**^ **Ala**_**Ac**_-encoded variants, herein termed ^**SSB**^ **T7-RNAP**, were successfully expressed, purified, and characterized by SDS-PAGE, MALDI-TOF mass spectrometry, CD, UV–vis, and fluorescence spectroscopy. Incorporation of ^**TCNB**^ **Ala**_**Ac**_ introduced distinct and enhanced fluorescence signatures while largely preserving the overall secondary structures of the engineered enzymes. Notably, these novel ^**TCNB**^ **Ala**_**Ac**_-encoded variants exhibited higher relative RNA yields than those of wild-type T7-RNAP under identical in vitro transcription conditions. Molecular docking further supported the structural compatibility of the modification with the promoter-bound enzyme. These findings establish GCE-based, site-specific incorporation of an FTUAA into a functionally relevant site of T7 RNA polymerase as a promising strategy for generating spectroscopically traceable, transcriptionally competent T7-RNAP variants for future enzyme engineering and mechanistic studies.

## Graphical abstract

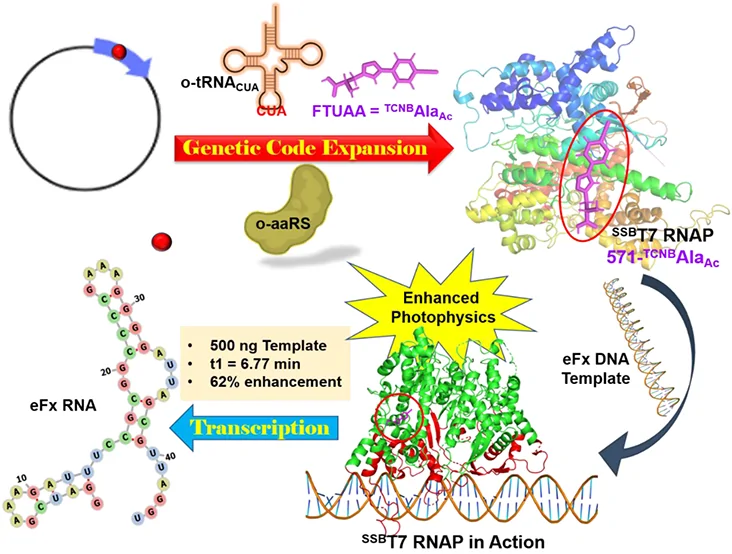

## Introduction

The bacteriophage T7 RNA polymerase (T7 RNAP) is a crucial enzyme in molecular biology and biotechnology.^1^ Unlike multisubunit RNA polymerases, the monomeric structure of T7 RNAP enables it to function independently of auxiliary transcription factors.^2,3^ Its high promoter specificity, robust RNA synthesis capability, and well-characterized mechanistic and structural features make it an ideal model for studying transcription mechanisms^4,5^ and a preferred tool in synthetic biology and biotechnological applications.^6,7^ However, its high specificity for its native promoter sequence makes it sensitive to mutations at key functional sites.^8,9^ One such residue is tyrosine 571 (Y571), which has been extensively investigated owing to its important role in transcription.^10^ Therefore, T7 RNA polymerase is often chosen as a model system mainly due to its well-characterized structure, high transcriptional efficiency, and widespread use in in vitro transcription platforms. Specifically, the Y571 residue represents a known functionally important residue, making it a valuable position to study and understand the effect of GCE-mediated incorporation of an unnatural amino acid on enzyme activity.

This site is located at the N-terminus of helix W, within a critical DNA-binding cleft near the catalytic pocket that is made up of residues Asp-537, Lys-631, Tyr-639, Gly-640, and Asp-812.^11,12^ Previous studies have demonstrated that Y571 plays a pivotal role in promoter recognition and stabilization of nucleotide triphosphates (NTPs) during RNA synthesis.^13^ However, mutational substitutions at this site, frequently observed as Y571S or Y571F, are known to disrupt transcription efficiency by altering substrate-binding specificity.^14,15^ Furthermore, undesired mutations are also known to compromise the fidelity of RNA synthesis by interfering with crucial hydrogen-bonding and aromatic-stacking interactions.^16,17^ These mutations ultimately lead to a significant loss of enzymatic activity or complete inactivation. The Y571S substitution, caused by a point mutation from UAC to UCC, was reported to result in impaired RNA synthesis due to the loss of key interactions in the active site.^17,18^ However, traditional mutagenesis approaches have failed to fully compensate for the structural and functional role of Y571.^19^ Collectively, all of these observations highlight the functional importance of Y571 and make it an attractive site for studying whether the incorporation of a chemically distinct unnatural amino acid (uAA) can be achieved while preserving the overall structural integrity and transcriptional competence of T7 RNAP.

To this end, genetic code expansion (GCE) technology offers a powerful approach for addressing functionally important residues by site-specific incorporation of uAAs with tailored chemical and functional properties (Figure 1A).^20^ This technique, pioneered by Schultz and colleagues,^21^ has enabled the precise introduction of uAAs into proteins using orthogonal tRNA/aminoacyl-tRNA synthetase (aaRS) pairs, thereby overcoming the limitations of traditional mutagenesis and chemical labeling methods.^22,23^ GCE also allows the introduction of novel functionalities into proteins, including fluorescence, photocrosslinking, and chemical reactivity.^23,24^ One particularly impactful application is substituting functionally important residues in proteins with uAAs to enhance their stability and functionality or introduce new properties without disrupting their native activities. uAAs with interesting photophysical properties, such as fluorescent triazolyl unnatural amino acids (FTUAAs), have gained much of our interest in protein engineering for installing/modulating intrinsic fluorescence property.^25^ Incorporating these uAAs into catalytically relevant regions can enable the modulation of enzymatic behavior while simultaneously allowing the introduction of intrinsic fluorescent probes for enhancement in photophysical characteristics.^26,27^

**Figure 1:**
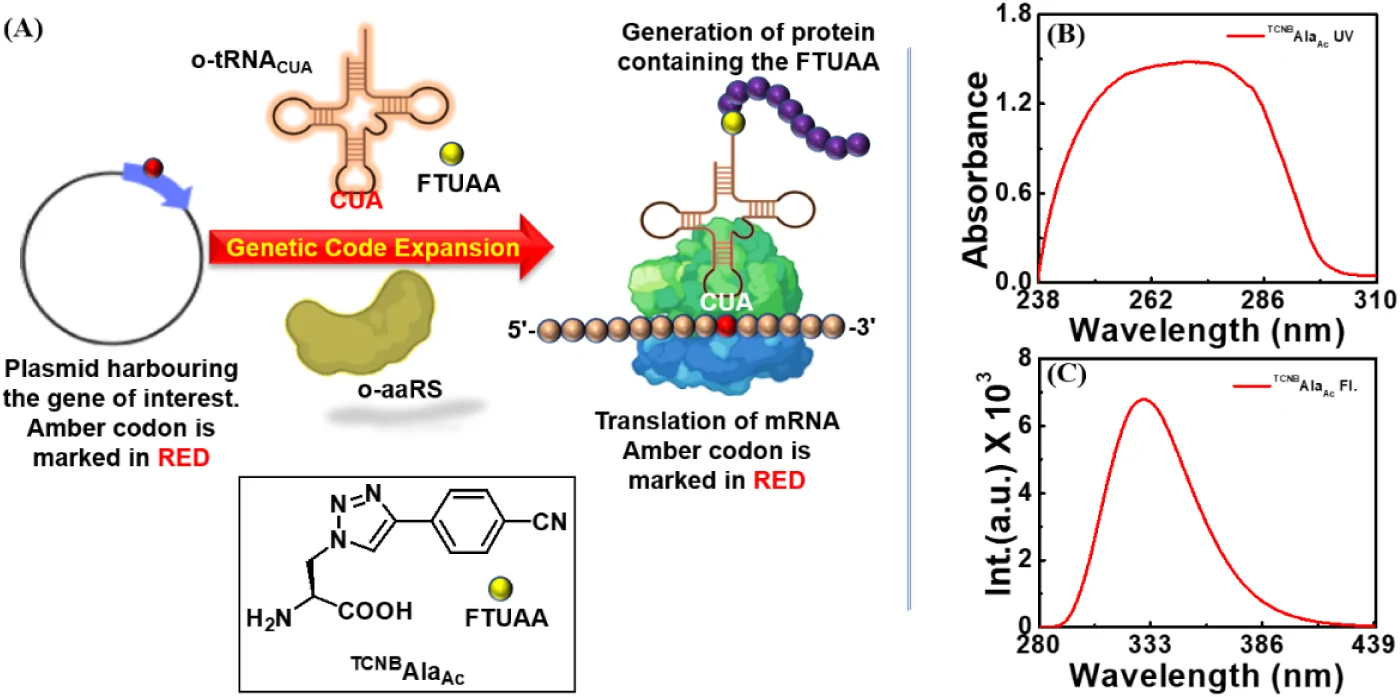
(A) The genetic code expansion (GCE) technology in general and the structure of the fluorescent triazolyl unnatural amino acid (FTUAA) **^TCNB^Ala_Ac_** used in this study for genetic encoding. (B–C) UV–visible and fluorescence (λ_ex_ = 270 nm) emission spectra of our **^TCNB^Ala_Ac_** (1 mM) in dialysis buffer.

Therefore, as part of our current research activity in the expansion of the genetic code and the design of fluorescent triazolyl unnatural amino acids, we sought to address a functionally important residue in T7 RNAP by substituting Y571 with a member of the FTUAA family, ^**TCNB**^ **Ala**_**Ac**_ (Figure 1).^28^ The logic behind choosing this amino acid is as follows. Recently, we have incorporated four fluorescent triazolyl unnatural amino acids, including ^**TCNB**^ **Ala**_**Ac**_ into GFP at the 150th position within the β-barrel structure. The results observed, particularly in the case of ^**TCNB**^ **Ala**_**Ac**_ demonstrating a FRET interaction with increased fluorescence brightness in the FTUAA-encoded sfGFPs, made it an ideal candidate for incorporation in T7 RNAP.^29^ Further, even though the FTUAA is structurally different from tyrosine, the presence of a polar −CN functional group may allow it to mimic the polarity of −OH in tyrosine with similar H-bonding opportunity, therefore possibly allowing it to participate in local interactions taking place in the protein microenvironment.^16,17^ Additionally, the aromatic triazolyl moiety, a trans-amide mimic, exhibits high stacking propensity, bioassociability via H-bonding, and has been reported to modulate the fluorescence photophysics of an attached, weakly fluorescent chromophore. The stacking propensity of the phenyl ring in tyrosine (Y571), thus, could possibly be mimicked by the more efficient triazolyl phenyl moiety of ^**TCNB**^ **Ala**_**Ac**_. Furthermore, the aromatic and hydrogen-bonding features of the ^**TCNB**^ **Ala**_**Ac**_ residue may allow it to participate in local interactions within the DNA-binding cleft.^30^ All these features, therefore, made ^**TCNB**^ **Ala**_**Ac**_ a suitable candidate for exploring the consequences of introducing a chemically distinct fluorescent residue at the functionally important Y571 position.

Therefore, in this work, we report the GCE-mediated incorporation of ^**TCNB**^ **Ala**_**Ac**_ at the 571st codon position of T7 RNAP using two orthogonal tRNA/aaRS pairs: pDule Tet3.0 ^31^ (A1 system) and pEvol-pylT-N346A-C348A (A2 system).^32^ Although both orthogonal translation systems, as expected, produced the same engineered RNA polymerase, here termed ^**SSB**^ **T7 RNAP**, we employed them to evaluate the overall efficiency of ^**TCNB**^ **Ala**_**Ac**_ incorporation using independently evolved amber codon suppression systems. The engineered enzymes were subsequently characterized with respect to their photophysical properties, structural integrity, and in vitro transcriptional ability. Importantly, all these studies were carried out independently to analyze the consequences of the GCE-mediated incorporation of ^**TCNB**^ **Ala**_**Ac**_ without implying a direct relationship between transcriptional behavior and the observed photophysical properties. Furthermore, molecular docking analysis was performed to gain further insight into the structural compatibility of uAA incorporation within the promoter-bound conformation of T7 RNAP and to provide a possible structural rationale for the experimental observations. Overall, this study aims to examine the consequences of ^**TCNB**^ **Ala**_**Ac**_ incorporation at a functionally important residue of T7 RNAP while providing a foundation for the future development of fluorescently engineered RNA polymerases.^33^

## Results and Discussion

To achieve our goal, we started our investigation by engineering the wild-type T7 RNA polymerase gene (WT-T7 RNAP) to site-specifically accommodate an amber stop codon (TAG) at position Y571. The modified gene was then cloned into a pET21a vector downstream of the T7 promoter region, allowing high protein expression levels in *Escherichia coli* (BL21 DE3 strain) (see SI, Section 3). Detailed experimental protocols, including construction of plasmids, site-directed mutagenesis (SDM), protein expression, and photophysical studies, are provided in the Supporting Information (see SI, Sections 2.1–2.10).^34^ Successful cloning was initially validated by colony PCR. A distinct DNA band near 2.6 kbp observed on a 0.7% agarose gel established the cloning of the T7 RNAP gene into the pET21a vector (See SI, Section 4, Figure S1). Further confirmation of gene cloning into the expression vector was achieved through restriction digestion studies using two restriction enzymes, *Bam*HI and *Eco*RI (See SI, Section 4, Figure S1). Additionally, incorporation of the amber codon mutation at position Y571 was confirmed using colony PCR (see SI, Section 4, Figure S2), followed by Sanger sequencing. Co-transformation of the mutant with the (a) T7 RNAP gene-containing plasmid together with (b) the o-aaRS/tRNA pair-bearing plasmid (A1/A2) into *E. coli* BL21-DE3 cells, followed by induction with IPTG and supplementation with 1 mM ^**TCNB**^ **Ala**_**Ac**_, resulted in the expression of ^**SSB**^ **T7 RNA** Polymerase using two independent orthogonal translation systems (A1: Tet3.0 o-aaRS and A2: pylT-N346A-C348A o-aaRS), wherein both produced the same encoded protein containing the ^**TCNB**^ **Ala**_**Ac**_ uAA at the 571st site.

The two orthogonal aaRS systems chosen for this study were used to decode the amber (TAG) stop codon, thereby allowing the incorporation of ^**TCNB**^ **Ala**_**Ac**_ at the 571st codon position. Herein, the use of two separate orthogonal translation systems was primarily intended to evaluate the overall reproducibility of uAA incorporation using two independently evolved o-aaRS/tRNA pairs rather than generate distinct protein variants. The SDS-PAGE analysis (Figure 2A) of the whole-cell lysates showed distinct protein bands near 100 kDa only in the presence of ^**TCNB**^ **Ala**_**Ac**_, thereby supporting full-length protein expression. As expected, in the absence of ^**TCNB**^ **Ala**_**Ac**_, the presence of the ectopic TAG codon led to premature termination of the translation process, preventing protein formation and indicating the dependence of the ^**SSB**^ **T7 RNAP** protein expression depends on GCE-mediated amber suppression. Additionally, the Tet3.0 orthogonal aaRS/tRNA pair (A1) employed in this study has previously been validated in our laboratory using an sfGFP-150TAG reporter system, whereby we recorded the formation of full-length sfGFP only in the presence of the corresponding unnatural amino acid, while no full-length protein was produced in its absence, as reported in our previous work.^29^ The A2 system also showed similar results (data not published) when used to express FTUAA-encoded sfGFP. Thus, all of these observations collectively highlight the fidelity of the orthogonal translation systems used for site-specific amber suppression.

**Figure 2:**
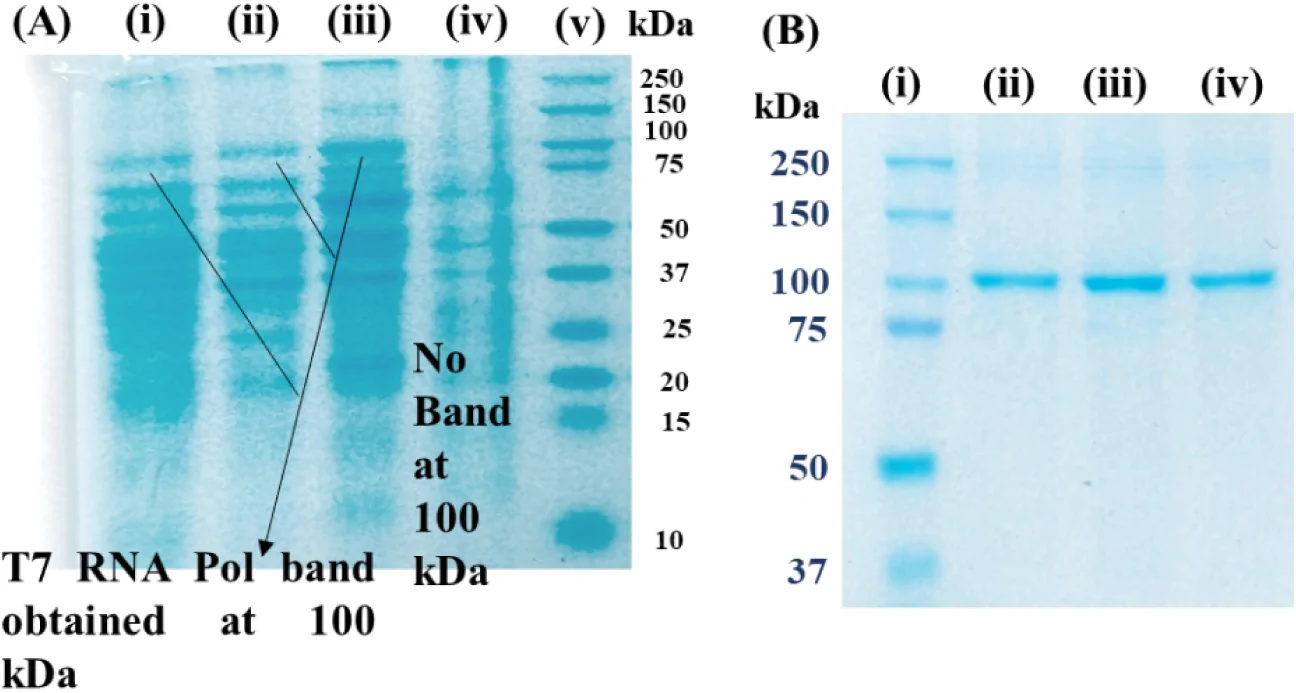
(A) SDS-PAGE analyses of the total cell lysates depicting the expression of wild-type (WT) and encoded T7 RNA polymerase variants (in the presence and absence of **^TCNB^Ala_Ac_**). Lane (i): WT-T7 RNAP; Lane (ii): **^SSB^T7 RNAP** expressed with the A1 orthogonal aaRS/tRNA system, in the presence of **^TCNB^Ala_Ac_**; Lane (iii): **^SSB^T7 RNAP** expressed with the A2 orthogonal aaRS/tRNA system, in the presence of **^TCNB^Ala_Ac_**; Lane (iv): **^SSB^T7 RNAP** expressed with the A1 orthogonal aaRS/tRNA system, in the absence of **^TCNB^Ala_Ac_**; Lane (v): molecular weight marker. A prominent protein band near 100 kDa for lanes (i-iii) indicates the successful expression of the WT and encoded T7 RNAPs. (B) SDS-PAGE gel image showcasing the protein bands of wild-type T7 RNAP protein and **^SSB^T7 RNAP** respectively purified using IMAC chromatography. Lane (i): molecular weight marker; Lane (ii): wild-type T7 RNA polymerase; Lanes (iii) and (iv): purified encoded **^SSB^T7 RNAP** proteins formed in the presence of **^TCNB^Ala_Ac_** and orthogonal aaRS systems (A1 = Tet3.0 and A2 = pylT-N346A-C348A), respectively. The amount of protein in each case in this figure was kept constant at 1 mg/mL.

Next, the expression products containing a His_6_-tag, were purified using nickel-affinity chromatography with Ni-NTA resin. Protein concentrations of the purified T7 RNAP variants were determined with the help of the Bradford assay. The WT protein depicted a yield of 3.5 mg per 100 mL of bacterial culture, whereas the ^**TCNB**^ **Ala**_**Ac**_ containing variants, A1 and A2, exhibited slightly lower, yet comparable protein yields of 2.9 mg and 3.1 mg per 100 mL, respectively. These results therefore indicate that the incorporation of ^**TCNB**^ **Ala**_**Ac**_ at codon position 571 does not substantially affect overall protein expression or purification under the experimental conditions employed. For further downstream analyses, all the purified protein samples were normalized to a concentration of 1 mg/mL prior to SDS-PAGE and the study of photophysical properties. The detailed quantitative data on protein yields derived from Bradford assay experiments are provided in Table S4 of the SI.

The purified proteins, normalized to a concentration of 1 mg/mL, were then analyzed using SDS-PAGE (Figure 2B). The WT-T7 RNAP showed a distinct protein band near ∼100 kDa on the SDS-PAGE gel when eluted with the elution buffer. Additionally, the loss of protein in the flow-through fraction and the fractions obtained after washing with the wash buffer was found to be minimal. The SDS-PAGE gel image of the wild-type protein purification is shown in Figure S5 of the Supporting Information. Comparably, the expressed ^**SSB**^ **T7 RNA** polymerase bands, formed by the incorporation of ^**TCNB**^ **Ala**_**Ac**_ with the help of the A2 system (see SI, Figure S6) and the A1 o-aaRS system (see SI, Figure S7) respectively, were found to be similar in nature to that of the wild-type T7 RNA polymerase. All the expressed proteins showed a band in SDS-PAGE close to the ∼ 100 kDa molecular weight marker, with high intensity appearing up to the second or third elution fractions.^35^

To further characterize ^**SSB**^ **T7 RNAP** and in order to obtain additional evidence to support the GCE-mediated incorporation of ^**TCNB**^ **Ala**_**Ac**_, into the full-length ^**SSB**^ **T7 RNAP**, MALDI-TOF mass spectra of the intact protein samples were recorded and analyzed. The monoisotopic molecular weights of all the proteins were calculated from their amino acid sequences using the Peptide Mass Calculator online server (https://www.peptidesynthetics.co.uk/tools/). The MALDI-TOF analysis confirmed that the experimentally observed molecular weights of all the purified WT and encoded T7 RNAP variants lie within ±1 Da of their calculated masses, and the differences between the observed and calculated molecular weights agreed well with the calibration error of the instrument (Table 1 and See SI, Section 6, Figure S8, Table S5).^36^ Thus, together with the amber-suppression-dependent protein expression observed by SDS-PAGE and the previously validated fidelity of the o-aaRS/tRNA pairs in earlier studies,^29,37,38^ the MALDI-TOF analyses provide complementary evidence consistent with the GCE-mediated incorporation of ^**TCNB**^ **Ala**_**Ac**_ into ^**SSB**^ **T7 RNAP**. Furthermore, the distinct UV–visible absorption and fluorescence characteristics discussed in the following sections also provide additional support consistent with the successful incorporation of ^**TCNB**^ **Ala**_**Ac**_ into ^**SSB**^ **T7 RNAP**.

**Table 1:** MALDI-TOF Mass Analysis of the Wild-Type and Encoded T7 RNA Polymerase

**Sl No.**	**Protein Name**	**aaRS Used**	**Calculated Monosiotropic MS (Da)**	**MALDI-TOF MS (Da)**
1	WT-T7 RNAP	-	101138.29	101138.91
2	^SSB^T7 RNAP	Tet3.0 (A1)	101214.31	101214.07
3	^SSB^T7 RNAP	pylT-N346A-C348A (A2)	101214.31	101213.73

After isolating and characterizing the pure ^**TCNB**^ **Ala**_**Ac**_-encoded ^**SSB**^ **T7 RNAP**, we next elucidated the secondary structures of the encoded enzymes using circular dichroism (CD) spectroscopy, and compared the results with those of the WT protein. It is noteworthy that in order to study the photophysical properties (using CD, UV, and fluorescence spectroscopy), all the purified and dialyzed encoded protein samples were adjusted to the same concentration (1 mg/mL) before recording the spectral data. Thus, far-UV CD spectra for the WT protein displayed the classical α-helical signature, with a strong positive band near 200 nm and two negative bands at 209 and 220 nm (Figure 3A). In contrast, both the encoded ^**SSB**^ **T7 RNAPs** also exhibited negative bands near 209 and 220 nm, but with lower intensity. They further exhibited two positive bands between 195 and 200 nm, as depicted in Figure 3A. From the CD spectra, we therefore observed that the FTUAA-encoded variants exhibited similar spectral features, while showing differences in band intensities. These differences likely reflect the local perturbations introduced by the incorporation of ^**TCNB**^ **Ala**_**Ac**_ at the 571st site. Thus, the overall secondary conformation appears to be largely preserved, indicating that the global folding of the encoded proteins is maintained. All these observations suggest that the incorporation of ^**TCNB**^ **Ala**_**Ac**_ does not cause major perturbations in the overall secondary structure of the engineered T7 RNAP. Next, we studied the UV–visible and fluorescence spectral properties of the enzymes.

**Figure 3:**
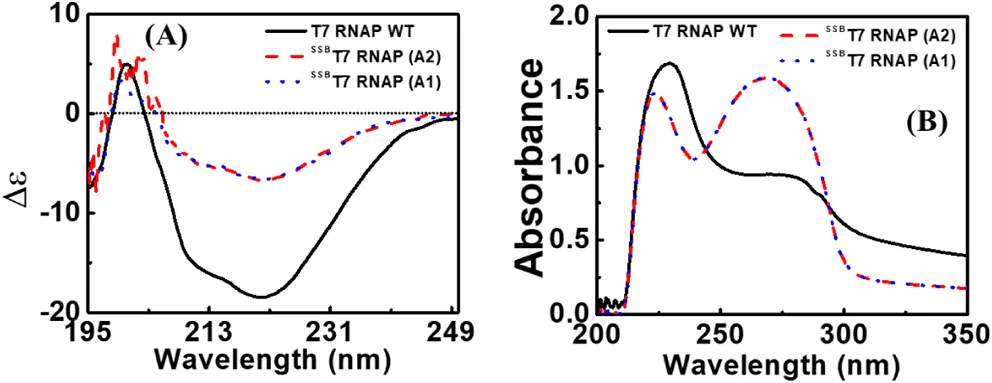
(A) CD and (B) UV–visible spectra of the wild-type and the **^TCNB^Ala_Ac_**-encoded T7 RNA polymerase enzymes [concentration = 1 mg/mL].

The UV–visible spectra, as shown in Figure 3B for the WT-T7 RNAP, show a primary weak absorption maximum near 279 nm, characteristic of intrinsic aromatic amino acids (tryptophan, tyrosine, and phenylalanine) present in any protein. A minor shoulder was also observed near 290 nm, which likely arose from π→π* transitions of tryptophan residues in asymmetric microenvironments, as reported and in line with the spectral signatures of α-helical proteins. Beyond 300 nm, the UV–vis spectrum declined steadily without any further distinct features. On the other hand, the ^**TCNB**^ **Ala**_**Ac**_-encoded ^**SSB**^ **T7 RNAP** variants exhibited a distinct absorption band with a maximum near 270 nm, extending up to 300 nm, which can be directly attributed to the absorption maximum of the incorporated ^**TCNB**^ **Ala**_**Ac**_ FTUAA (Figure 3B). The overlap of the 270 nm band with the intrinsic aromatic absorption of the protein was also evident; however, a unique and strong absorption peak at 270 nm distinguishes the encoded enzyme from the UV–vis spectral data of the WT-T7 RNAP. The FTUAA chromophore used in this study is known to strongly absorb near 270 nm (as shown in Figure 1B), and the formation of a distinct absorption band near this region is consistent with the presence of an incorporated ^**TCNB**^ **Ala**_**Ac**_ residue in the encoded ^**SSB**^ **T7 RNAP** variants.

Another interesting feature observed in the UV–vis spectra of the encoded enzymes was that the relative intensities of the ^**TCNB**^ **Ala**_**Ac**_-derived band at 270 nm and the intrinsic aromatic absorption band at 279 nm appeared additive in nature, eventually resulting in an overall enhancement of absorbance in the 260–290 nm wavelength range. The encoded enzymes also exhibited a hypsochromic shift of ∼10 nm in the absorption maximum region, compared to WT-T7 RNAP, suggesting that alterations in local electronic properties are introduced upon incorporation of the uAA. Since the spectra of both ^**SSB**^ **T7 RNAP** variants appeared to be nearly identical, we suggest that the observed spectral changes with respect to the WT protein are attributable primarily to the GCE-mediated site-specific incorporation of ^**TCNB**^ **Ala**_**Ac**_, rather than to the o-aaRS/tRNA pair systems (A1 and A2) used for protein expression.

Encouraged by the distinct absorbance features, we then analyzed the fluorescence emission profiles of the WT and encoded T7 RNAPs (Figure 4A–C). Based on the UV–vis spectra, the excitation wavelengths were chosen as 270, 280, and 290 nm, respectively. Across all excitation wavelengths, WT-T7 RNAP exhibited a single, weak emission band at approximately 333 nm. In contrast, the ^**SSB**^ **T7 RNAPs** exhibited a broad and strong emission band (Figure 4). The broad band exhibited an intense shoulder at around 330–333 nm and a maximum with strong intensity at around 360–370 nm. Next, the emission spectra of the ^**SSB**^ **T7 RNAPs** were deconvoluted and resolved into two different components (Figure 5) using a Lorentzian–Gaussian fitting approach. The overlapping emission profiles were resolved into two distinct components, and only fits satisfying χ^2^ < 5 were considered acceptable for further studies (see **ESI**). The resolved components were then further used to evaluate the contribution of the individual emission features to the overall fluorescence profile. Herein, we observed that the 330–333 nm emission profile appeared to be consistent with the fluorescence characteristics of the incorporated ^**TCNB**^ **Ala**_**Ac**_ FTUAA, whereas the origin of a second band centered around 365 nm is discussed below.

**Figure 4:**
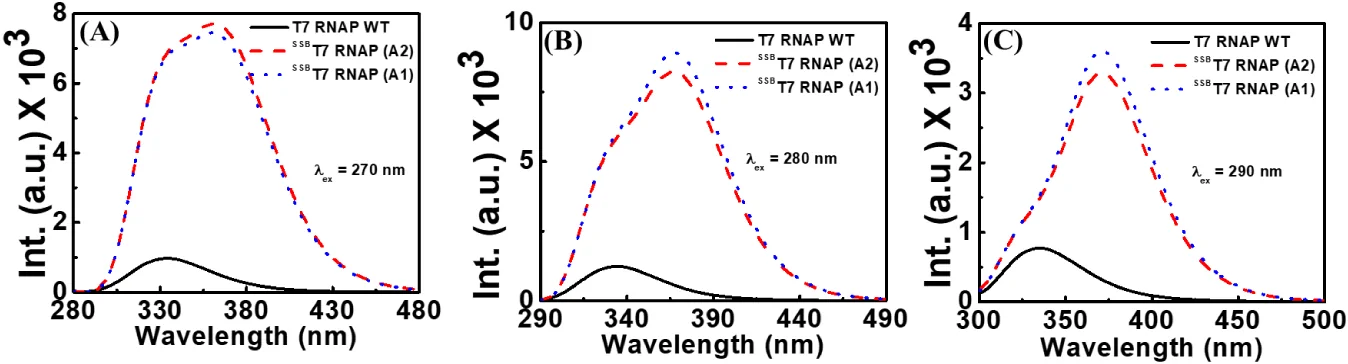
Fluorescence spectra of the wild-type and the **^TCNB^Ala_Ac_**-encoded ^SSB^T7 RNA polymerase enzymes at various excitation wavelengths. λ_ex_ = (A) 270 nm; (B) 280 nm; and (C) 290 nm [concentration = 1 mg/mL].

**Figure 5:**
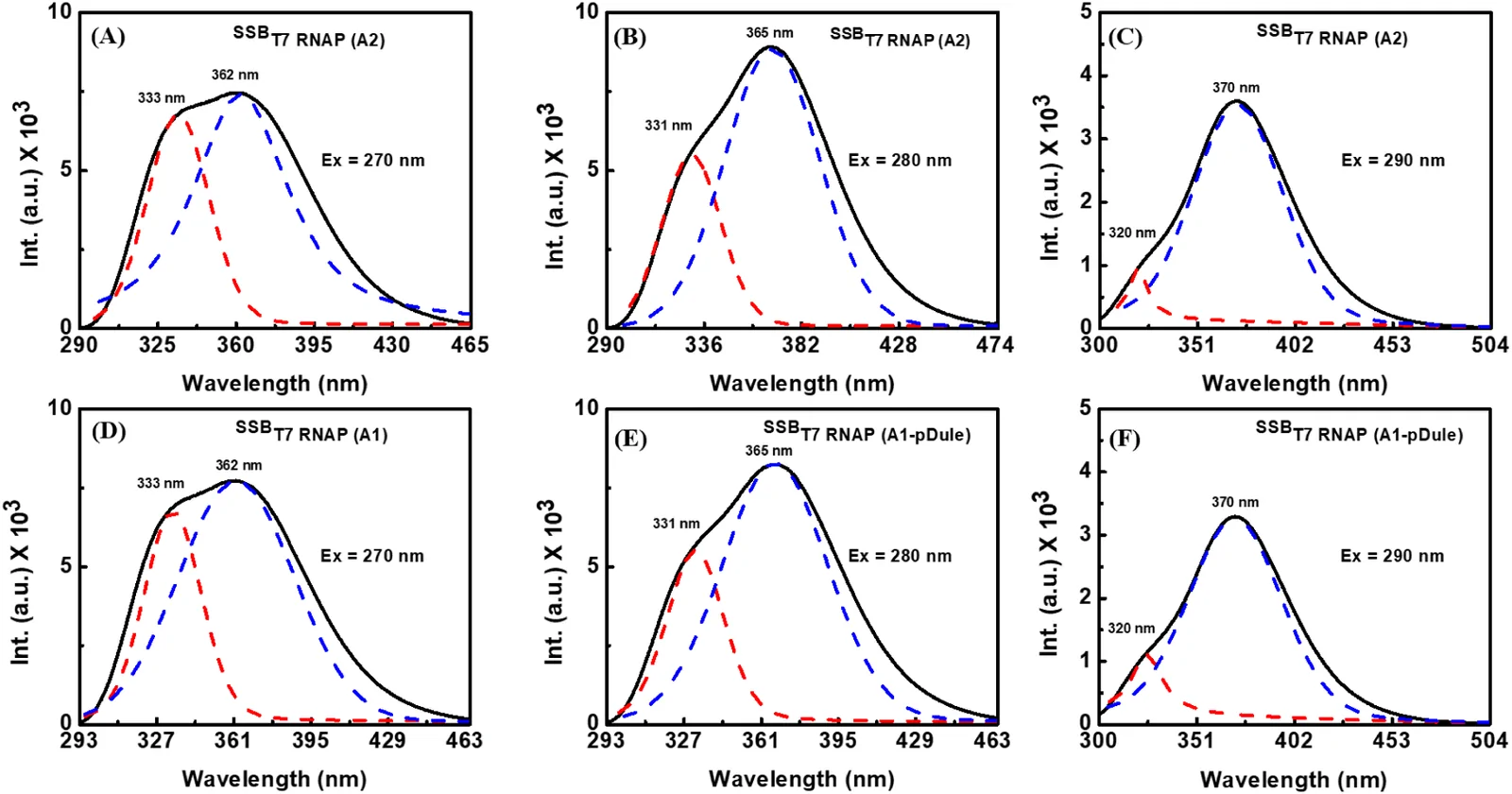
Resolved (into two bands) fluorescence spectra of the **^TCNB^Ala_Ac_**-encoded ^SSB^T7 RNA polymerase enzymes at various excitation wavelengths. λ_ex_ = (A) and (D): 270 nm; (B) and (E): 280 nm; and (C) and (F): 290 nm [concentration = 1 mg/mL].

It is noteworthy that the intensities of both emission bands were found to be almost identical when excited at 270 nm. However, the intensity of the band at 330–333 nm decreases when excited at 280 nm, and is even less intense when excited at 290 nm. In contrast, the decrease in intensity of the band at 365 nm is considerably less pronounced. The appearance of the long-wavelength emissive band at 365 nm may be explained as follows. The free ^**TCNB**^ **Ala**_**Ac**_ amino acid is known to exhibit an intrinsic emission band centered around approximately 330–333 nm in dialysis buffer. Consequently, the generation of the bathochromically shifted emission pattern recorded near 365 nm in ^**SSB**^ **T7 RNAP** suggests that the photophysical behavior of the uAA is influenced by its local protein microenvironment (Figure 5). A plausible reason for this may be the formation of local electronic interactions between the incorporated ^**TCNB**^ **Ala**_**Ac**_ residue and its neighboring aromatic amino acid residues, such as tryptophan, tyrosine, and phenylalanine, which may in turn alter the excited-state properties of the chromophore, thereby giving rise to an additional emission band. This enhanced fluorescence intensity may similarly reflect the modulation of the uAA residue by the surrounding protein environment. Therefore, although the formation of an exciplex arising from π-π stacking interactions represents a potential explanation for our observations, future spectroscopic and photophysical investigations will be valuable for establishing the underlying mechanism while also elucidating the exact excited-state interactions primarily responsible for the observed emission properties.

Taken together, the fluorescence data suggest that although ^**TCNB**^ **Ala**_**Ac**_ is naturally fluorescent near 330 nm, its incorporation into T7 RNAP using the GCE technique results in the generation of new emission states not visible in the free amino acid or from tryptophan in buffer. The appearance of a bathochromically shifted band between 360 and 370 nm in the encoded proteins is consistent with the possibility that the ^**TCNB**^ **Ala**_**Ac**_ participates in electronic interactions with adjacent aromatic amino acid residues present in the local microenvironment, possibly through exciplex formation via π-π stacking interactions. To understand the origin of emission at 365 nm, variable-temperature fluorescence was recorded. Thus, when ^**SSB**^ **T7 RNAP** was excited at the absorption maximum of incorporated FTUAA ( $\lambda_{max}^{abs}$ = 270 nm; ^**TCNB**^ **Ala**_**Ac**_), the fluorescence intensity of the 365 nm band gradually decreased with an increase in temperature from 20 to 40 °C^39^ while the 330 nm band experienced a gradual enhancement of intensity, supporting the possibility of π-π stacked exciplex emission at 365 nm (Figure 6). Interestingly, a gradual increase in temperature did not lead to any change in intensity for the band at 330 nm when excited at 280 nm (data not shown), corresponding to the absorption of tryptophan. All these data suggest that the incorporated ^**TCNB**^ **Ala**_**Ac**_ residue is mainly responsible for the observed temperature-dependent emission characteristics, rather than the intrinsic tryptophan residues. The results further indicate that the uAA-encoded protein’s local microenvironment influences the excited-state behavior of the incorporated fluorophore, which may in turn contribute to the formation of the additional emission band centered around 365 nm. Our interpretation is further supported by the corresponding excitation spectra.

**Figure 6:**
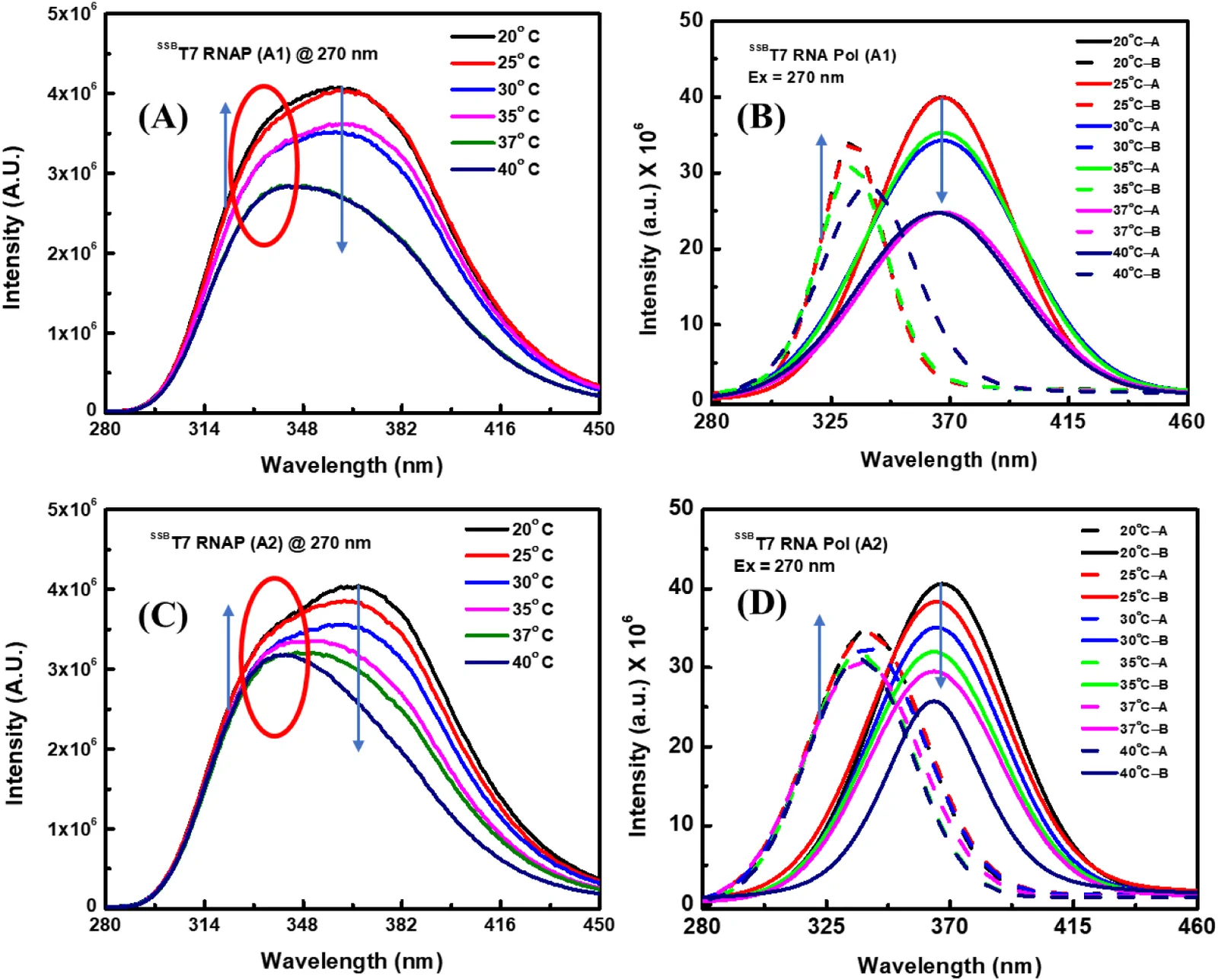
Variable-temperature fluorescence spectra of **^TCNB^Ala_Ac_**-encoded **^SSB^T7 RNA** polymerase enzymes at excitation = 270 nm, consistent with the possibility of exciplex emission. (A) Original and (B) resolved spectra using the A1 system. (C) Original and (D) resolved spectra using the A2 system.

Based on our findings, we next evaluated the fluorescence quantum yields (Φ_f_) and fluorescence brightness to further establish electronic communication between the native aromatic residues and the FTUAA used in this study. The WT-T7 RNAP recorded low Φ_f_ values of ∼ 0.096, 0.099, and 0.076 when excited at wavelengths of 280, 270, and 290 nm, respectively. As expected, the overall fluorescence brightness of the wild-type protein at these wavelengths was lower, consistent with the intrinsically weak fluorescence of tryptophan. On the other hand, the encoded ^**SSB**^ **T7 RNAP** variants displayed significantly higher values of Φ_f_. The A1 variant recorded Φ_f_ values of 0.55, 0.58, and 0.40 when excited at 280, 270, and 290 nm, respectively, whereas the A2 variants recorded similar Φ_f_ values of 0.53, 0.62, and 0.43 when excited at the same wavelengths, respectively. The overall fluorescence brightness (ε × Φ_f_) values were also found to be 10 times higher in comparison to the WT-T7 RNAP (SI, Table S6). Such enhancements in quantum yield and fluorescence brightness values underscore the primary role of the ^**TCNB**^ **Ala**_**Ac**_ residue as a radiative center.

Therefore, all the observations with regard to photophysics suggest that an overall improvement in emission properties results from the incorporation of ^**TCNB**^ **Ala**_**Ac**_ via the GCE strategy. The resolution of emission spectra into distinct dual-band features validates the additive contributions from the native aromatic amino acids and ^**TCNB**^ **Ala**_**Ac**_, with the FTUAA accounting for the recorded enhancement in quantum yield and fluorescence brightness. The proteins were also visualized under UV light (305 nm), wherein we observed that the encoded ^SSB^T7 RNA polymerase samples were highly fluorescent and exhibited a bluish color (see SI, Figure S9). The fluorescence intensity for the wild-type enzyme, however, was not very prominent. Our findings, therefore, highlight the potential role of the GCE technique as a versatile strategy for engineering target enzymes with tailored fluorescence properties through the site-specific incorporation of FTUAAs. The unique photophysical characteristics of ^**SSB**^ **T7 RNA polymerase** thus provide a strong foundation for future studies aimed at the development of fluorescent polymerases for investigating protein conformational dynamics, monitoring enzymatic processes, and further expanding the applications of genetically encoded, intrinsically fluorescent enzymes in biophysical and biochemical research.

Following our promising photophysical results, we evaluated the transcriptional ability of our encoded ^**SSB**^ **T7 RNAPs** through in vitro transcription assays.^40^ To this end, the eFx^41^ DNA template (sequence: 5′-GTAATACGACTCACTATAGGATCGAAAGATTTCCGCGGCCCCGAAAGGGGATTAGCGTTAGGT-3′; T7 promoter region underlined) was generated with the help of a template-independent multistep PCR strategy.^42^ The logic behind choosing the eFx DNA sequence was that it contains a T7 promoter region, which would allow the native T7 RNA polymerase to bind at the onset of transcription, as evidenced by Suga and colleagues.^43^ Additionally, the eFx DNA sequence has been regularly used for genetic reprogramming strategies using flexizymes.^44^ Therefore, we felt that it would be worthwhile to evaluate the transcriptional ability of our encoded ^**SSB**^ **T7 RNA** polymerase on the eFx DNA template. Subsequently, the eFx construct was selected as a representative model template^45,46^ to facilitate a direct comparison of the overall transcriptional performance of WT T7 RNA polymerase and ^**SSB**^ **T7 RNA** polymerase under identical experimental conditions.

The formation of the eFx DNA was confirmed by electrophoresis on an ethidium bromide (EtBr)-stained 3% agarose gel (Figure S10, Supporting Information). The resulting eFx DNA was then purified and utilized as the template for transcription reactions mediated by the encoded ^**SSB**^ **T7 RNAP** variants harboring the ^**TCNB**^ **Ala**_**Ac**_ FTUAA at the 571st codon position, incorporated by the A1 and A2 systems, respectively. Simultaneously, a control in vitro transcription reaction was performed with the WT RNAP enzyme.^42^ The products of transcription were then resolved on an EtBr-stained 12% denaturing polyacrylamide gel, which validated the successful generation of the anticipated eFx RNA products (Figure 7). From the gel, we noted that the RNA products generated with the encoded ^**SSB**^ **T7 RNAP** variants exhibited intensities greater than that of the RNA product obtained with the WT enzyme. These findings demonstrate that, upon GCE-mediated incorporation of ^**TCNB**^ **Ala**_**Ac**_, the ability of ^**SSB**^ **T7 RNAPs** to synthesize the expected RNA product remained preserved under identical experimental conditions. Furthermore, the consistently higher RNA band intensities observed for the engineered enzymes suggested greater relative RNA production with respect to the wild-type protein. However, even though these observations clearly suggest that the incorporation of ^**TCNB**^ **Ala**_**Ac**_ is compatible with efficient in vitro transcription, the present endpoint assay does not distinguish between the individual steps of transcription (such as promoter binding, initiation, elongation, or enzyme turnover). It may be noted that all reactions are expected to generate RNA products of identical length; however, minor differences in band appearance are possible due to gel resolution and/or staining variability. Encouraged by these outcomes, we proceeded further to investigate the transcriptional behavior of the modified enzymes.

**Figure 7:**
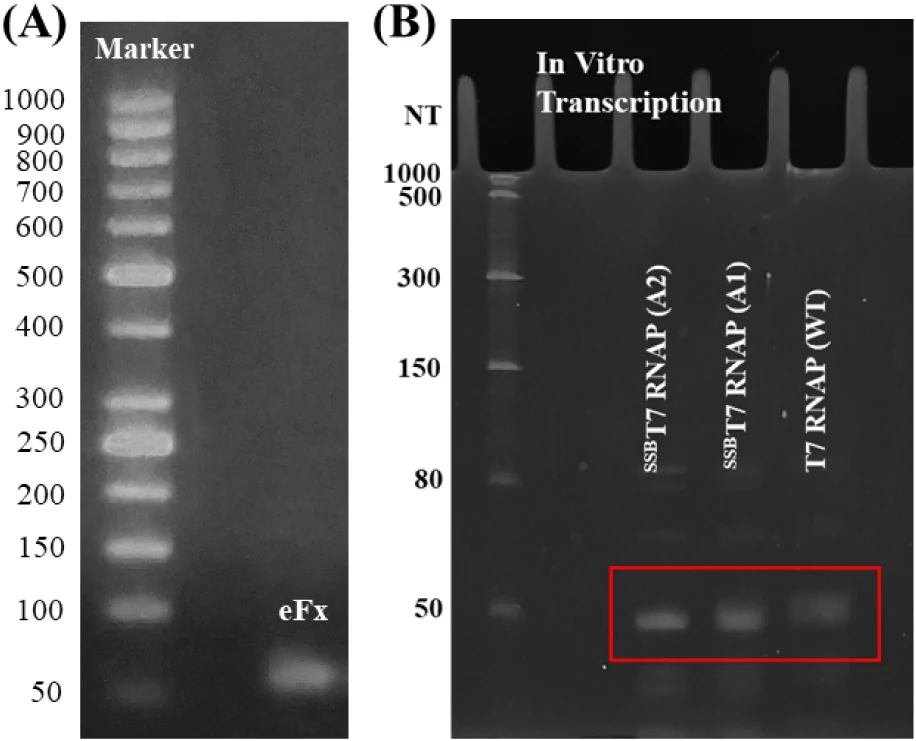
(A) Visualization of the eFx DNA template on an EtBr stained 3% agarose gel. (B) Visualization of RNA bands of eFx obtained after in vitro transcription reactions using the encoded T7 RNA polymerase enzymes (**^SSB^T7 RNAP** containing **^TCNB^Ala_Ac_** incorporated by A1 and A2 orthogonal aaRS/tRNA pairs, respectively) and the WT-T7 RNA polymerase enzyme (positive control).

The transcriptional performance of the encoded and WT enzymes was assessed by monitoring the time-dependent accumulation of RNA products at three different DNA template concentrations: (i) 100 ng, (ii) 500 ng, and (iii) 1000 ng (corresponding to the total amount of DNA added per reaction). These concentrations were selected to represent low (100 ng), intermediate (500 ng), and high (1000 ng) template regimes, commonly used in in vitro transcription (IVT) reaction setups. The range of DNA template concentrations was kept consistent with standard T7 RNAP-based IVT setups, which facilitated the assessment of RNA accumulation over a broad range of substrate availability while maintaining comparable reaction conditions.^47^

All reaction setups were monitored over a period of 240 min, and the resulting RNA products obtained at different intervals were quantified by densitometric analysis of the resolved RNA bands on 12% denaturing urea–polyacrylamide gels (Figure 8A–C). All experiments were performed in triplicate under identical conditions. The time-dependent RNA accumulation was determined by standard densitometric analysis of RNA gel bands using ImageJ software. The values thus recorded represent the relative RNA yields [(expressed in arbitrary units (au)], corresponding to the intensity of the RNA band depicting the expected eFx RNA transcript, and were used for comparative analysis between the native and the encoded enzymes. Furthermore, the data were fit using a single-exponential model, wherein the fitted parameter represents an apparent characteristic time constant (t_1_), highlighting the amount of RNA accumulation under the experimental conditions described in the **ESI**. The generated data sets enabled the quantitative analysis of the relative RNA accumulation profiles of the encoded and WT-T7 RNAP enzymes over the course of the transcription reactions. The minor additional RNA bands observed in the gels likely correspond to truncated transcripts, which have been previously recorded in T7 RNAP-based in vitro transcription reactions.^48,49^ Therefore, the major RNA band corresponding to the expected transcript was used for the quantitative analysis.

**Figure 8:**
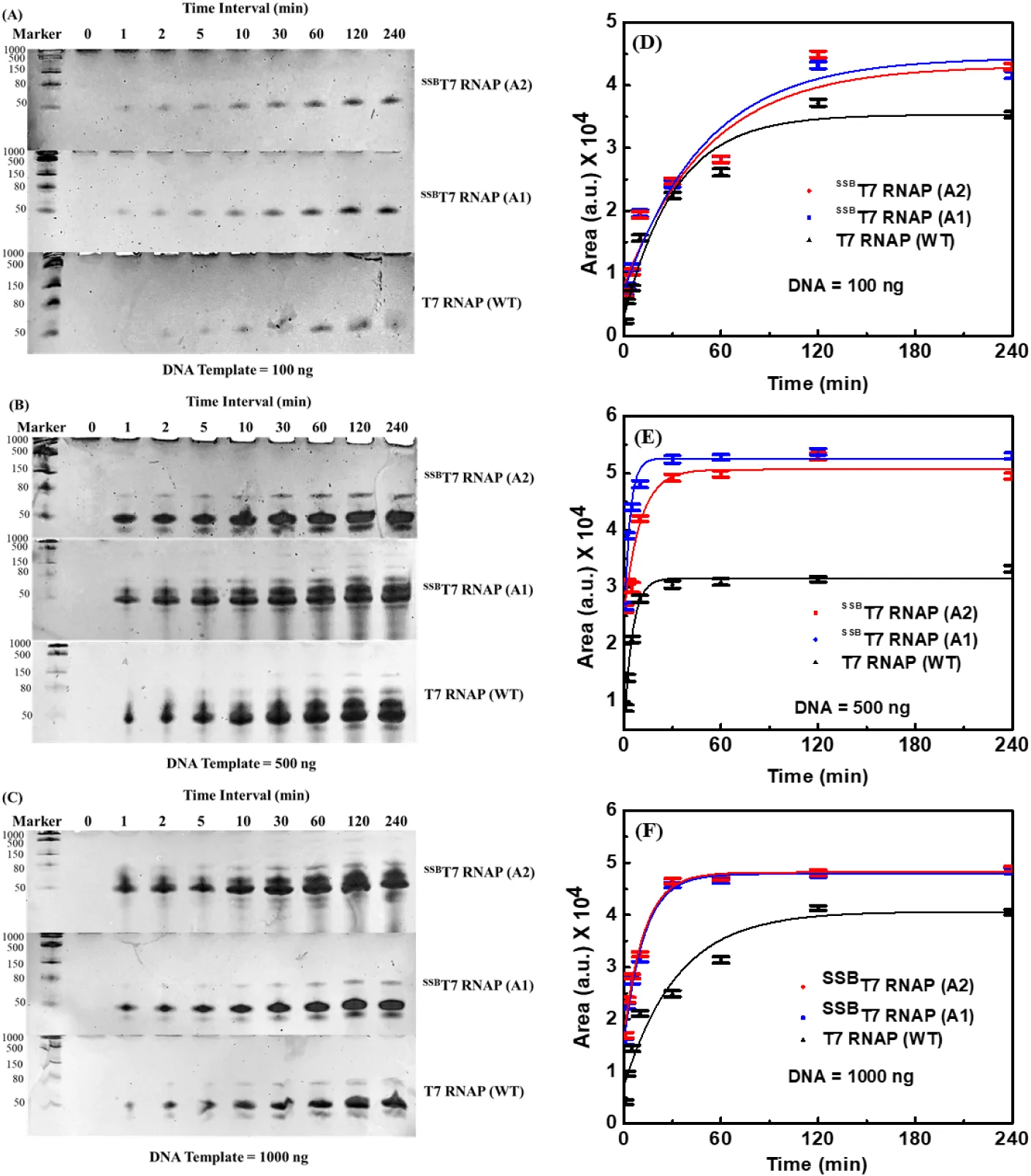
(A–C) Urea-PAGE gel depicting the formation of RNA by the wild-type T7 RNA polymerase and the engineered **^SSB^T7 RNA polymerases,** respectively, at various time intervals to study the relative RNA yields. The DNA template for each reaction setup was varied as follows: (A) eFx DNA template = 100 ng; (B) eFx DNA template = 500 ng; (C) eFx DNA template = 1000 ng. (D–F) The fitted RNA accumulation curves showing the RNA yields produced by the **^TCNB^Ala_Ac_**-encoded and wild-type T7 RNA polymerase enzyme variants at various eFx DNA template concentrations. eFx DNA template concentration for (D) = 100 ng; for (E) = 500 ng; and for (F) = 1000 ng. The data represent the mean ± standard error (SE) obtained from three independent experiments. The error bars indicate the standard error of the mean.

At the intermediate DNA template concentration of 500 ng (RNA gel depicted in Figure 8B), the native enzyme displayed a reduced relative RNA yield of 31,786 ± 538 au, along with an apparent characteristic time constant (t_1_) of 5.21 ± 0.62 min (Adj. R^2^ = 0.989). Strikingly, the ^**SSB**^ **T7 RNAP** variants achieved a relative RNA yield approximately 62% higher than that of the wild type. The encoded enzymes averaged a y_0_ value of 51,623 au (52,573 ± 1,205 au for system A1 and 50,673 ± 1,178 au for system A2). The fitted apparent time constants appeared to differ between the two expression systems, with an average t_1_ of 6.77 min (3.52 ± 1.06 min for A1; 10.03 ± 2.47 min for A2; Adj. R^2^ averaging 0.941). The overall differences in t_1_ values recorded between the data sets produced by the two different o-aaRS systems may be due to variations in the RNA accumulation profiles obtained under the respective experimental conditions. However, both support the general enhancement of relative RNA yields generated by the encoded ^**SSB**^ **T7 RNAP** enzyme over the wild type. The corresponding fitted RNA accumulation curves at 500 ng of DNA template concentration are shown in Figure 8E.

Next, at a DNA template concentration of 100 ng (RNA gel depicted in Figure 8A), the wild-type enzyme yielded a maximum relative RNA formation (y_0_) of 35,323 ± 2,326 au, and the apparent characteristic time constant (t_1_) was recorded as 31.56 ± 8.15 min (Adj. R^2^ = 0.962). In contrast, the encoded enzymes demonstrated a 24% increase in relative RNA yield, averaging a yield of 43,760 au (44,415 ± 3,685 au for the system A1-generated enzyme and 43,104 ± 3,727 au for the system A2-generated enzyme). The fitted RNA accumulation curves at 100 ng template concentration are shown in Figure 8D. However, both encoded enzymes generated from the two o-aaRS systems (A1 and A2) showed a slower apparent time constant, with t_1_ averaging 50.62 min (49.94 ± 15.01 min for system A1 and 51.29 ± 15.94 min for system A2). The difference in the apparent time constants between the native and the ^**TCNB**^ **Ala**_**Ac**_-encoded enzymes points toward distinct RNA accumulation profiles under the limiting template conditions employed. Despite this, the 24% enhancement in relative RNA yield suggests that the incorporation of the triazolyl unnatural amino acid is compatible with sustained RNA production, even when subjected to low-template transcription conditions. Overall, this feature may be of interest for future investigations requiring higher relative RNA yields^50^ at low-template ranges.

Finally, at a high template concentration of 1000 ng, the WT enzyme recorded a relative RNA yield of 40,623 ± 2,386 au and displayed an apparent characteristic time constant (t_1_) value of 32.88 ± 11.45 min (Adj. R^2^ = 0.939). On the other hand, the encoded ^**SSB**^ **T7 RNAP** demonstrated an average relative yield of 48,026 au (47,865 ± 1,018 au for system A1 and 48,186 ± 1,041 au for system A2). This observed relative yield was approximately 18% higher than that obtained using the WT enzyme. Additionally, the average apparent time constant for the encoded enzymes was recorded as 12.51 min (12.53 ± 2.15 min for system A1 and 12.49 ± 2.14 min for system A2; Adj. R^2^ averaging 0.98), as depicted in the respective figures. The overall fitted RNA accumulation curves of the RNA yields at 1000 ng template concentration are shown in Figure 8F, and the RNA gel image is depicted in Figure 8C. Overall, at the high template concentration studied, differences in the apparent time constants were observed between the WT and the encoded enzymes. These differences may reflect distinct RNA accumulation profiles under the higher template conditions employed. Despite this, the encoded enzymes exhibited an approximately 18% higher relative RNA yield than the WT enzyme under these conditions, further highlighting that the GCE-mediated incorporation of ^**TCNB**^ **Ala**_**Ac**_ is compatible with RNA production across the template concentrations examined.

Altogether, the time-dependent RNA accumulation profiles investigated at 100, 500, and 1000 ng DNA template concentrations consistently depicted higher relative RNA yields for the ^**TCNB**^ **Ala**_**Ac**_-encoded ^**SSB**^ **T7 RNAP** variants with respect to the WT enzyme, corresponding to approximately 24%, 62%, and 18% increases, respectively. All of these results support that the GCE-mediated incorporated ^**TCNB**^ **Ala**_**Ac**_ is compatible with sustained transcriptional activity across the range of template concentrations examined. Even though the apparent characteristic time constants were observed to vary between (i) the WT and encoded enzymes and (ii) the two orthogonal suppression systems, these differences may eventually reflect variations in the RNA accumulation profiles under the respective experimental conditions. The data presented in this work therefore provide a comparative assessment of RNA production, while future studies will help clarify how individual steps of transcription, including promoter binding, initiation, promoter escape, elongation, enzyme stability, and the active enzyme fraction, contribute to the observed RNA accumulation behavior. Nevertheless, the consistent increase in relative RNA production across all three template concentrations observed in our work highlights the overall compatibility of ^**TCNB**^ **Ala**_**Ac**_ incorporation with functional T7 RNAP activity, and also demonstrates an additional consequence of genetic code expansion in the engineered polymerase. Interestingly, the 500 ng DNA template concentration produced the highest relative RNA yields among the three template concentrations evaluated. This observation indicates a concentration-dependent variation in RNA accumulation under these defined experimental conditions, although the underlying basis of this behavior remains to be established. Furthermore, the transcription assays were performed using a single, well-established DNA template in order to facilitate a direct comparison between the wild-type and encoded variants. Assessment across multiple types of DNA templates may provide further insight into the generality of our observations and will be considered for future studies.

Finally, to support the observed transcriptional performance and to assess whether incorporation of the ^**TCNB**^ **Ala**_**Ac**_ FTUAA affects T7 promoter recognition, a molecular docking study was performed using the encoded ^**SSB**^ **T7 RNA** polymerase and the eFx DNA template. The encoded ^**SSB**^ **T7 RNA polymerase** containing the ^**TCNB**^ **Ala**_**Ac**_ residue at the 571st codon position was prepared using the PyMOL software, which was then processed using UCSF Chimera (version 1.16). All water molecules present in the PDB file were removed in order to avoid non-native clashes during molecular docking. The protein was then preprocessed using the software’s built-in DockPrep module to add missing hydrogens and assign atomic charges and was subjected to energy minimization involving 100 steps of steepest descent followed by 100 steps of conjugate gradient optimization, with a step size of 0.02 Å in both cases. None of the atoms were fixed during the energy minimization step, thus allowing complete relaxation of local torsions around the encoded FTUAA (^**TCNB**^ **Ala**_**Ac**_). The eFx DNA template containing a T7 promoter region was prepared with the help of the SCFBio IIT Delhi Nucleic Acid Builder (https://scfbio-iitd.res.in/software/drugdesign/bdna.jsp) online tool. The resulting double-stranded B-DNA was then checked for proper base pairing and helical twist, and finally, the backbone geometry was analyzed. The DNA model was then subjected to an energy minimization process, following the same method as mentioned previously. The energy-minimized protein and DNA molecules were then docked using the HDock server^51–53^ (http://hdock.phys.hust.edu.cn/), which is known to employ a hybrid template-based and ab initio algorithm specifically optimized for studying macromolecular interactions. A total of one hundred docked structures were generated by the HDock server for each protein sample, of which the structure with the most favorable docking score was chosen for further studies.

The highest-ranked molecular docking model generated by the HDock server (docking score: −214.51 kcal/mol) revealed a favorable and well-oriented, molecular-level interaction between ^**SSB**^ **T7 RNA polymerase** and the eFx DNA template, containing a T7 promoter region (Figure 9A). Therefore, the result obtained supports the formation of a potentially favorable binding interface between the encoded ^**SSB**^ **T7 RNA polymerase** and the DNA. The duplex DNA structure was further observed to occupy the standard promoter recognition cleft of the enzyme, displaying a binding geometry similar to that reported for the experimentally solved crystal structure of a WT-T7 RNA polymerase-T7 promoter complex (PDB ID: 1CEZ).^54^ A control molecular docking was performed using WT-T7 RNAP and the same eFx DNA sequence (Figure 9B) wherein the HDock score was found to be −205.36 kcal/mol. Nonetheless, even though the encoded variant exhibited a more favorable docking score in comparison to its wild-type counterpart, the docking scores alone do not constitute experimental evidence of enhanced promoter binding or increased transcriptional activity. However, these computational results do suggest that the site-specific incorporation of ^**TCNB**^ **Ala**_**Ac**_ is structurally well tolerated and compatible with T7 promoter recognition without introducing steric perturbations.

**Figure 9:**
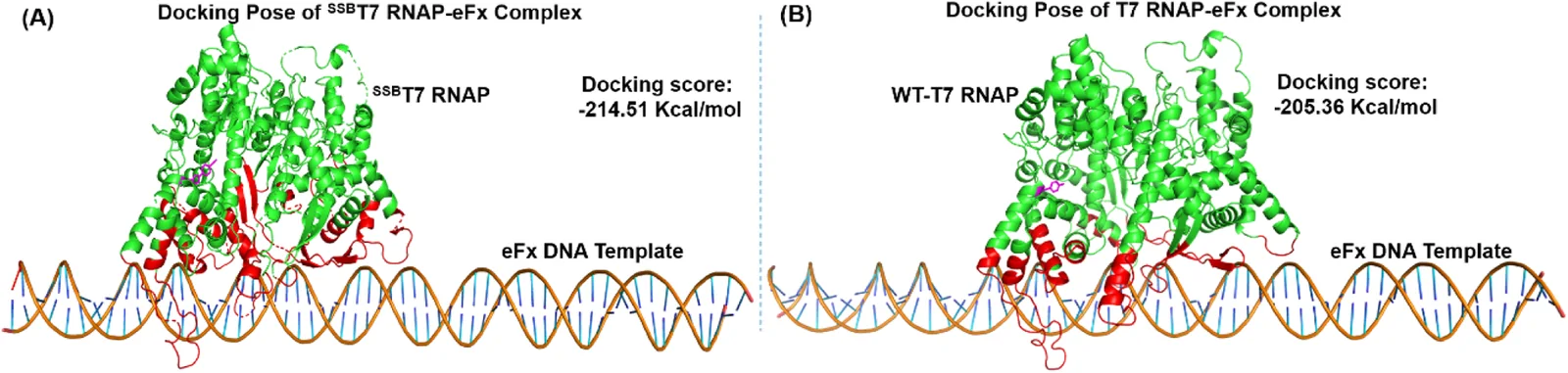
Best docking poses of T7 RNA polymerase bound to the eFx DNA template. (A) is for **^SSB^T7 RNA polymerase** and (B) is for WT-T7 RNA polymerase. The modified **^TCNB^Ala_Ac_** residue in **^SSB^T7 RNA polymerase** and tyrosine (Tyr) in WT-T7 RNA polymerase at position 571 have been depicted in magenta. The amino acid residues interacting with the eFx DNA template have been shown in red. The rest of the protein is shown in green in all cases.

Furthermore, the best docking pose indicated that the encoded FTUAA, ^**TCNB**^ **Ala**_**Ac**_, at residue 571 did not interfere with the overall positioning of the promoter region (See SI, Figure S11). The incorporated uAA remained oriented away from the DNA backbone without any apparent steric clash with the predicted promoter-binding geometry. No large distortions were observed in the N- or C-terminal domains, and the catalytic cleft also appeared to remain intact. These in silico observations therefore suggest that the incorporated ^**TCNB**^ **Ala**_**Ac**_ residue is structurally compatible with the overall architecture of T7 RNAP while also preserving the predicted promoter-binding geometry. This observation is also supported by the preservation of the overall domain arrangement and the absence of an apparent alteration in the position of the promoter with regard to the recognition elements of the polymerase. In this regard, the FTUAA used in this study may possibly serve as an intrinsic fluorescent probe in the future, suitable for monitoring local changes in the protein microenvironment during transcription, such as those associated with conformational transitions or promoter binding. However, this potential application was not explored in the present study and therefore remains to be experimentally established.

Taken together, the molecular docking studies provide an overall structural perspective on the effects of the site-specific incorporation of ^**TCNB**^ **Ala**_**Ac**_ at the 571st position. The predicted binding geometry indicates that, upon incorporation of FTUAA (^**TCNB**^ **Ala**_**Ac**_), no major structural changes within the promoter-recognition region of T7 RNA polymerase are introduced. The more favorable docking score observed for the encoded ^**SSB**^ **T7 RNAP** further provides an interesting basis for future investigation, although its relationship with T7 promoter recognition and the enhanced RNA production observed experimentally remains to be established. Nevertheless, the molecular docking studies complement the biochemical and photophysical findings by suggesting that the GCE-mediated incorporation of ^**TCNB**^ **Ala**_**Ac**_ is structurally compatible with the native architecture of T7 RNA polymerase. In this context, future studies involving direct promoter binding measurements and detailed transcriptional analyses will help elucidate these relationships and provide deeper insight into the molecular effects of ^**TCNB**^ **Ala**_**Ac**_ incorporation.

## Conclusion

We report the generation of highly fluorescent ^**SSB**^ **T7 RNAPs** using two orthogonal tRNA synthetase/tRNA systems to incorporate a fluorescent triazolyl unnatural amino acid, ^**TCNB**^ **Ala**_**Ac**_, at the Y571 site using a genetic code expansion strategy. The strong fluorescence emission of the ^**SSB**^ **T7 RNAP** variants may potentially arise from exciplex emission involving π-stacking interactions between the incorporated ^**TCNB**^ **Ala**_**Ac**_ residue and neighboring aromatic amino acid residues; a feature not observed in the wild-type enzyme. Coupled with these unique photophysical properties, the encoded enzymes also produced more prominent RNA transcripts and exhibited higher relative transcriptional yields, with a maximum enhancement of approximately 62% compared to the WT-T7 RNAP under identical experimental conditions. Thus, our findings demonstrate the overall compatibility of the triazolyl FTUAA with functional T7 RNAP activity while introducing distinct photophysical characteristics into the enzyme. Nevertheless, further investigations involving detailed promoter binding and transcriptional analyses will help elucidate the molecular basis of these findings. Finally, this work also highlights the potential of the GCE strategy for developing state-of-the-art active polymerases and other enzymes with unique photophysical characteristics for probing enzyme behavior and future applications in biotechnology and synthetic biology.

## Materials and Methods

### Cloning of the T7 RNA Polymerase Gene into the pET21a Vector and Site-Directed Mutagenesis (SDM)

The T7 RNA polymerase gene was amplified from the *E. coli* BL21­(DE3) genome using gene-specific primers and cloned into the pET21a vector following restriction digestion with *Bam*HI and *Eco*RI restriction enzymes. Subsequently, site-directed mutagenesis was performed to introduce an amber stop codon (TAG) at the Y571 position. The pET21a-T7 RNAP 571TAG construct, thus obtained, was cotransformed into *E. coli* BL21­(DE3) cells with two different orthogonal aminoacyl-tRNA synthetase/tRNA systems: pDule-Tet3.0 (A1) and pEVOL-pylT-N346A/C348A (A2). The DNA sequences of the primers used in this study are listed in Tables S1–S2 (Supporting Information).

### Protein Expression Studies

Next, the expression of the engineered T7 RNA polymerase, designated ^SSB^T7 RNAP, was carried out in the presence of 1 mM ^**TCNB**^ **Ala**_**Ac**_, a fluorescent triazolyl unnatural amino acid (FTUAA), to facilitate its incorporation at the Y571 position. For this, transformed *E. coli* BL21 (DE3) cells were grown in Luria–Bertani (LB) broth supplemented with the appropriate antibiotics at 37 °C and 180 rpm. Secondary cultures were inoculated with 2% (v/v) of the respective overnight cultures and grown until an OD_600_ of approximately 0.5. Protein expression was induced with 2 mM l-arabinose and 1 mM IPTG, followed by the addition of 1 mM uAA. These cultures were further incubated for 6 h, after which the cells were harvested by centrifugation at 6000 rpm for 20 min at 4 °C. The cell pellets were then resuspended in chilled lysis buffer (50 mM Tris-HCl, 100 mM NaCl, 1 mM imidazole, pH 8.0) and lysed by probe sonication under cold conditions. The crude lysates were centrifuged at 10,000 rpm for 30 min at 4 °C, and the resulting supernatants were collected for protein purification.

The expressed proteins containing a C-terminal His_6_-tag were purified using immobilized metal affinity chromatography with Ni-NTA resin. The resin-bound proteins were initially washed with wash buffer (50 mM Tris-HCl, 0.5 M NaCl, 10 mM imidazole, pH 8.0), followed by elution using an elution buffer containing 400 mM imidazole. Subsequently, the eluted protein fractions were dialyzed for 24 h against chilled phosphate buffer (50 mM PB, 100 mM NaCl, pH 8.0), with the buffer replaced every 6 h. Protein concentrations were determined using the Bradford assay, with bovine serum albumin as the standard, and the protein samples were analyzed by 10% SDS-PAGE following denaturation at 95 °C for 10 min. Further details are provided in the Supporting Information.

### Circular Dichroism Spectroscopy

All purified and dialyzed protein samples were normalized to 1 mg/mL for subsequent analysis of secondary structure using CD spectroscopy. Circular dichroism (CD) spectra were recorded using a J-1500 spectropolarimeter (Jasco) equipped with a temperature-controlled unit and a 0.1-cm path length quartz cuvette. The spectra were acquired from 200 to 600 nm at 25 °C with three accumulations at a scan rate of 100 nm/min under continuous nitrogen purging.

### Photophysical Studies

The purified and dialyzed proteins were normalized to a concentration of 1 mg/mL for the subsequent spectroscopic characterization. UV–visible absorption spectra were recorded using a Shimadzu UV–visible spectrophotometer over a wavelength range of 200–800 nm. Fluorescence emission spectra were recorded using a Hitachi F-7000 fluorescence spectrophotometer with a slit width of 5 nm, using the absorption maxima as the excitation wavelengths. Fluorescence spectra were deconvolved using XPSPeak41 software, and the quantum yields and fluorescence brightness were calculated by using the respective reference standards. Details are provided in the Supporting Information.

### MALDI-TOF Mass Analysis

The wild-type and recombinant ^SSB^T7 RNA polymerase protein samples were purified following the protocols mentioned previously. The eluted samples were dialyzed using a dialysis buffer (50 mM PB, 100 mM NaCl, pH 8.0) to get rid of excess imidazole and glycerol present in the protein elution buffer. The MALDI-TOF spectra of the protein samples were analyzed using a sinapinic acid matrix on a Bruker Autoflex Speed. The matrix for sample analysis was prepared by dissolving 1 μg of sinapinic acid in 50 μL analyte solution (0.1% TFA dissolved in Milli-Q H_2_O). This mixture was further combined in a 1:1 ratio with HPLC-grade acetonitrile to obtain the final matrix. The samples for MALDI-TOF analysis were prepared in various protein:matrix ratios, such as 1:1, 1:2, 1:5, and 1:10. The instrument comprises a MALDI ionization source along with a TOF/TOF mass analyzer. Bruker Daltonics FlexAnalysis software was used to obtain and visualize the spectra.

### In Vitro Transcription Reaction

The DNA template containing the T7 promoter region was prepared using a multistep overlapping PCR method. Next, in vitro transcription reactions were performed using the purified wild-type and engineered T7 RNA polymerases, following previously reported protocols. The reaction mixtures were then incubated at 37 °C for 4 h, followed by DNase I treatment at the same temperature for 30 min. The RNA products were recovered by phenol-chloroform extraction, followed by ethanol precipitation. The purified RNA samples were denatured in RNA loading buffer and resolved using 12% denaturing urea-PAGE. The RNA bands were visualized after ethidium bromide staining using a UV transilluminator.

For transcription kinetics studies, the purified DNA templates were quantified using a NanoDrop spectrophotometer at 260 nm. The transcription reactions were performed at a constant final enzyme concentration of 0.1 mg/mL using 100, 500, and 1000 ng of DNA template. Each transcription reaction mixture was incubated at 37 °C, and 20 μL aliquots were withdrawn at the time points 0, 1, 2, 5, 10, 30, 60, 120, and 240 min. The reactions were then quenched using a stop buffer containing 8 M urea and 50 mM EDTA. The RNA products were resolved by denaturing urea-PAGE, and the band intensities were quantified using ImageJ. The background-subtracted intensities were normalized against the RNA ladder, and the resulting data were plotted and fitted to a one-phase association model using OriginPro 2025. All experiments were performed in triplicate, and standard errors were calculated accordingly. Further details are provided in the Supporting Information.

### Molecular Docking Studies

Molecular docking studies were performed to investigate the structural compatibility of the FTUAA-encoded ^SSB^T7 RNAP with the eFx DNA template. The protein structure was prepared by using PyMOL and UCSF Chimera (version 1.16), followed by structural refinement and energy minimization. The eFx DNA duplex was generated using the SCFBio IIT Delhi Nucleic Acid Builder and similarly energy-minimized. Protein–DNA docking was performed using the HDock server under default parameters. The resulting docked structures were analyzed using PyMOL and Discovery Studio to examine DNA positioning, domain orientation, and possible intermolecular interactions.

Detailed experimental procedures, including primer sequences, plasmid construction, purification conditions, spectroscopic analyses, and docking parameters, are provided in the Supporting Information.

## Supplementary Material



## Data Availability

All experimental procedures and spectroscopic data can be found in the Supporting Information (SI).

## Notes

General Experimental Protocols, Cloning and Site-Directed Mutagenesis, Plasmid Maps, Protein Yield and SDS-PAGE Gel Electrophoresis Images, MALDI-TOF Mass, Photophysical Studies, In Vitro Transcription Studies, Docking Poses and Various Interactions, ^1^H NMR Spectra of the Fluorescent Triazolyl Unnatural Amino Acid (PDF)
