# Supplementary material for: Genetically Expanded Fluorescent SSBT7 RNA Polymerase in Action: Intriguing Photophysics with Enhanced RNA Production
Source: ACS Omega. 2026 Oct 15;11(44):68791–803. doi: 10.1021/acsomega.6c07950 (PMC13757076; doi:10.1021/acsomega.6c07950)
Supplement: Supplementary file 1 [file ao6c07950_si_001.pdf]

[SUPPLEMENTARY INFORMATION]

Genetically Expanded Fluorescent ^{SSB}T7 RNA Polymerase in Action: Intriguing Photophysics with Enhanced RNA Production

Subhendu Sekhar Bag,[*,a,b,c] Aniket Banerjee[b] and Sinchini Barman[b]

[a]Chemical Biology/Genomics Laboratory, Department of Chemistry, [b]School of Interdisciplinary Studies and Sustainability, [c]JBM School of Health Science and Technology, Indian Institute of Technology Guwahati, Guwahati, India-781039. Email: ssbag75@iitg.ac.in

Sl. No.	Topics	Page No.
1.	**Materials and Methods**	1
2.	**General Experimental Protocols**	1-7
3.	**Cloning and Site-Directed Mutagenesis**	8
4.	**Plasmid Maps**	9-10
5.	**Protein Yield and SDS-PAGE Gel Electrophoresis Images**	10-12
6.	**MALDI TOF Mass Spectrometry**	12-13
7.	**Photophysical Studies**	13-14
8.	**In Vitro Transcription Studies**	14
9.	**Docking Poses and Various Interactions**	15
10.	**^{1}H NMR Spectra of the Fluorescent Triazolyl Unnatural Amino Acid**	15-16
11.	**References**	16-17

1. Materials and Methods

The pEVOL-pylT-N346A/C348A (A2 system) was a gift from Wenshe Liu[1] while the pDule-Tet3.0 (A1 system) was a gift from Ryan Mehl.[2] All the PCR reactions were conducted using PCR master mix procured from Promega. Additionally, the NucleoSpin Gel and PCR Clean-up kit was obtained from Machery-Nagel, whereas the PureYield™ Plasmid Miniprep System for plasmid isolation was obtained from Promega. The Q5 site-directed mutagenesis kit was purchased from New England Biolabs. The fluorescent unnatural amino acid $^{\mathbf{TCNB}}\mathbf{Ala_{Ac}}$ was synthesized in our laboratory and used for the incorporation studies using a genetic code expansion approach. The details of the other chemicals and reagents have been provided in the Experimental Protocols section.

2. General Experimental Protocols

2.1. Cloning of the T7 RNA Polymerase Gene into pET21a Vector and Site-Directed Mutagenesis

The T7 RNA polymerase gene was amplified using standard PCR techniques, utilizing the whole genome of BL21 DE3 strain of *E. coli* as the template, and T7 RNA polymerase gene specific primers (listed in **Table S1**). The pET21a vector and the amplified gene were then subjected to double digestion reactions using BamHI and EcoRI restriction enzymes. This was followed by agarose gel electrophoresis of the double restriction enzyme-digested products followed by the extraction of the DNA from the gel. The DNA samples were then ligated using T4 DNA ligase (Promega) following standard protocols. The ligated product was then transformed to chemically competent *E. coli* DH5α cells and were subsequently plated on ampicillin plates and kept in an incubator maintained at 37°C for overnight. The positive clones were then selected for subsequent site-directed mutagenesis (SDM) reaction. The SDM was done by following the manufacturer's instructions, using specific primers, the sequences of which have been listed in **Table S2**. These primer sequences were generated using the NEBaseChangerTool (https://nebasechanger.neb.com/).[3] After SDM, the mixture was again transformed in chemically competent *E. coli* DH5α cells and grown on selective media plates kept at 37°C for overnight. The positive clones, verified by DNA sequencing, were then grown in LB broth containing ampicillin (100 µg/ml) and the resulting plasmid, namely pET21a-T7 RNAP 571TAG was isolated using the PureYield™ Plasmid Miniprep System (Promega) for the development of orthogonal tRNA/tRNA synthetase systems.

Table S1: Primers used for the amplification of the T7 RNA Polymerase gene.

Primer	Sequence
T7-BamHI (FW)	5'-CGGGATCCATGAACACGATTAACA-3'
T7-EcoRI (Rev)	5'-CGGAATTCTTACGCGAACGCGAAG-3'

Table S2: Primers used for the introduction of TAG Stop codon at the 571st codon position T7 RNA Polymerase gene using site-directed mutagenesis (SDM) technique.

Primer	Sequence
T7-SDM (FW)	5'-AGGACATCTAGGGGATTGTTG-3' (Mutated base underlined)
T7-SDM (Rev)	5'-GAACGGTTTCACTAGGAAG-3'

2.2. Development of Orthogonal tRNA/tRNA Synthetase Systems for Incorporation of FTUAA ($^{TCNB}Ala_{Ac}$) into the Gene of Interest

Plasmids were isolated from two different cells of the *E. coli* DH5α strain; (i) one harbouring the pET21a-T7 RNAP 571TAG plasmid and (ii) the other harbouring the pDule Tet3.0 plasmid following the manufacturer's protocol (Promega). Chemically competent *E. coli* BL21-DE3 cells were then initially transformed with pET21a-T7 RNAP 571TAG plasmid (Amp^R) and were allowed to grow overnight at 37°C on LB plates containing 100 µg/ml ampicillin. The transformants were selected and a fresh batch of chemically competent cells of the BL21-DE3 cells already containing the pET21a-T7 RNAP 571TAG was prepared using standard protocol. These cells were then transformed with the pDule Tet3.0 (Tet^R) plasmid and were allowed to grow overnight at 37°C on ampicillin (100 µg/ml) and tetracycline (10 µg/ml) dual antibiotic plates. The positive transformants were then selected for studying the site-directed incorporation of fluorescent triazolyl unnatural amino acid **$^{TCNB}Ala_{Ac}$** into **^{SSB}T7 RNA polymerase** enzyme.

A similar approach was followed for the pEvol-pylT-N346A-C348A (Cmp^R) aaRS system, to generate BL21 DE3 cells harbouring dual plasmid encoding for the engineered T7 RNA polymerase and the pylT-N346A-C348A tRNA/tRNA synthetase pair. These transformed colonies were grown on ampicillin (100 µg/ml) and chloramphenicol (25 µg/ml) dual antibiotic LB plates.

2.3. Study of Incorporation of $^{TCNB}Ala_{Ac}$ in ^{SSB}T7 RNA Polymerase Expressed in *E. coli*

E. coli BL21-DE3 cells harbouring the pET21a-T7 RNAP 571TAG and the pDule Tet3.0 plasmids (A1) respectively were allowed to grow in a sterile test tube containing 3ml Luria Bertani (LB) broth supplemented with 100 µg/ml ampicillin and 10 µg/ml tetracycline in a shaker incubator set at 37°C, 180 rpm for overnight. Similarly, *E. coli* BL21-DE3 cells harbouring the pET21a-T7 RNAP 571TAG and the pEvol-pylT-N346A-C348A (A2) plasmids respectively were allowed to grow in a sterile test tube containing 3ml Luria Bertani (LB) broth supplemented with 100 µg/ml ampicillin and 25µg/ml chloramphenicol in a shaker incubator set at 37°C, 180 rpm for overnight. 100 ml secondary cultures for each variant containing 2% inoculum from their respective primary bacterial cultures were prepared and allowed to grow in a shaker incubator set at 37°C, 180rpm until the desired cellular densities were reached (OD_{600} ~ 0.5). The 2° cultures were then taken out and induced with 2 mM L-arabinose and 1mM IPTG followed by the addition of 1 mM FTUAA (**$^{TCNB}Ala_{Ac}$**). The bacterial cultures were then further incubated under pre-set conditions for 6 hours. A control experiment was also set up following similar experimental conditions except for no FTUAA being supplied.

Post induction, the broth containing the bacterial cells was transferred to 50 ml sterile Falcon tubes and centrifuged in a refrigerated centrifuge (HERMLE Z327K) set at 6000 rpm, 4°C for 20 minutes. The supernatant was then discarded and the cell pellet was lysed with chilled lysis buffer (50 mM Tris HCl, 100 mM NaCl, 1 mM imidazole, pH = 8.0). This was followed by probe sonication of the lysed cells under cold conditions using a probe ultrasonicator (Make: Sonics vibra-cell) set at 29 Amp, Pulse: 3 s, De-pulse: 5 s. After sonication, the crude lysate is subjected to another round of centrifugation, wherein the parameters were set as 10000 rpm, 4°C for 30 minutes. Post centrifugation, the supernatant was separated from the cell pellet and both of them were analysed on SDS-PAGE gel to confirm the site-directed incorporation of the $^{\mathbf{TCNB}}\mathbf{Ala}_{\mathbf{Ac}}$ FTUAA in the engineered T7 RNA polymerase protein.

2.4. Purification of the Encoded T7 RNA Polymerase Containing the FTUAA at the 571st Position

The cells were again regrown following the process mentioned previously. The cellular supernatant was then taken and subjected to purification using the immobilized metal affinity chromatography (IMAC) technique. A purification column (Make: BioRad) was washed with MilliQ water for 10 times followed by charging the column with 1ml of nickel-nitrilotriacetic acid (Ni-NTA) resin. The resin was then subjected to multiple washing steps using MilliQ water. The supernatant obtained after cell lysis was then loaded into the column, mixed well, and incubated at 4°C over a rocker, overnight. Post-incubation, the unbound cell lysate was collected as a flow-through, and the bound protein was subjected to further purification steps. The column containing the bound protein was initially washed 5 times using 10 ml wash buffer (50 mM Tris HCl 0.5 M NaCl, 10 mM imidazole, pH = 8.0), with an incubation time of 15 minutes between each wash cycle. After completing the washing steps, 1 ml elution buffer (50 mM Tris HCl 0.5 M NaCl, 400 mM imidazole, pH = 8.0) was added and the protein was collected in microcentrifuge tubes (MCTs) and stored at 4°C. The elution step was repeated for 8-9 times, with an incubation period of 30 minutes between each elution cycle. The protein samples eluted from the column were finally dialyzed using LA395-110 dialysis membrane (Make: HiMedia) and chilled dialysis buffer (50 mM PB, 100 mM NaCl, pH = 8.0). The dialysis was carried out for 24 hours, with the buffer being replaced with fresh buffer after every 6 hours. The purified, desalted protein samples obtained after dialysis were then stored in a refrigerator held at 4°C for further characterization and analysis. The temperature for all the washing, elution, dialysis, and storage steps was fixed at 4°C. Wash buffers with higher imidazole concentrations (50 mM) were not found to be suitable, as it led to protein loss in the washing steps. Therefore, an optimum imidazole concentration of 10 mM was used in all the washing steps.

2.5. Total Protein Concentration Analysis and SDS-PAGE

The total protein concentration was measured by following the protocol for a standard Bradford assay. Bovine Serum Albumin (BSA) (Loba Chemie) was used for setting the standard curve, and the measurements were taken using a microplate reader (Multiskan GO, Thermo Scientific) set at a fixed wavelength of 595nm. The protein concentrations were further cross-verified using a Nanodrop spectrophotometer (Implen NanoPhotometer N80) at a wavelength of 280nm (A_{280}).

The T7 RNA polymerase protein samples were then analysed using SDS-PAGE. Towards this end, the samples were mixed with the sample loading buffer (0.6 M Tris-HCl; pH = 6.8, 0.5 % SDS, 0.05% Bromophenol blue, 10% sucrose, 0.5% β-mercaptoethanol) in a ratio of 2:1 (v/v). They were then mixed well and subjected to heat at 95°C for 10 minutes to ensure the complete denaturation of proteins. The protein samples were subsequently resolved using 10% SDS PAGE gel with the help of a BioRad Mini Protean Electrophoresis System. Precision Plus Protein™ Dual Color, Bio-Rad make was used as the reference marker.

After gel electrophoresis, the SDS-PAGE gel was stained using a staining solution (0.1% Coomassie brilliant blue (Himedia), 50% Methanol (Finar) and 10% Glacial acetic acid (Himedia) dissolved in deionized water) for 30 minutes under shaking condition. Post-staining, the gel was destained using a destaining solution (50% methanol, and 10% acetic acid dissolved in deionized water). Once the excess dye was removed, the gel was taken out to identify the desired protein bands on the gel.

2.6. (A) Photophysical Study

The UV-visible spectra of the dialyzed T7 RNA polymerase protein samples (concentration fixed at 1 mg/ml) were measured using a UV-visible spectrophotometer (Shimadzu) across a wavelength range of 200-800 nm. The λ_{max} value was noted down and subsequently used as the excitation wavelength to record the fluorescence spectra of the protein samples, using a fluorescence spectrophotometer (Hitachi Fluorescence Spectrophotometer F-7000, slit width 5nm).

The following equation was used to calculate the quantum yield:

$$\Phi_S = \Phi_R \frac{Fl_S^{Area}}{Fl_R^{Area}} \frac{Abs_R}{Abs_S} \frac{n_S{}^2}{n_R{}^2}$$

where ΦR is the quantum yield of a standard reference, Fl_S^{Area} (sample) and Fl_R^{Area} (reference) are the integrated emission peak areas, Abs_S (sample) and Abs_R (reference) are the absorbance values at the excitation wavelength, and n_S (sample) and n_R (reference) are the refractive indices of the solutions.

The fluorescence emission spectra were deconvoluted using the XPSPeak41 software. A Shirley-type background was applied, followed by peak fitting using a Lorentzian-Gaussian function. The peak positions were fixed based on the experimentally observed spectral maxima, while the full width at half maximum (FWHM), peak area, and Lorentzian-Gaussian contribution were allowed to vary during the fitting procedure. The fitted peaks were subsequently optimized, and only fits with χ^2 values below 5 were considered acceptable.

(B) CD Spectroscopy Study

The purified protein samples were then scanned using a J-1500 spectropolarimeter (Jasco) equipped with a thermoelectric cooling-based temperature control unit in a 0.1 cm path length quartz cuvette, for measuring the CD spectra. Scanning was acquired as an average of three accumulations from wavelength 600 to 200 nm at a rate of 100 nm/min. Data integration time was 1 s under uninterrupted purging of nitrogen gas at a rate of 8.3×10^{-3} l/s at 25 °C. The obtained data were then plotted using Origin 2025 software.

2.7. MALDI TOF Mass Analysis

The wild-type and recombinant ^{SSB}T7 RNA polymerase protein samples were purified following the protocols mentioned previously. The eluted samples were dialyzed using a dialysis buffer (50 mM PB, 100 mM NaCl, pH = 8.0), to get rid of the excess imidazole and glycerol present in the elution buffer used to elute the protein. The MALDI-TOF spectra of the protein samples were analysed using a Sinapinic acid matrix on a Bruker Autoflex Speed Device. The matrix for sample analysis was prepared by dissolving 1 µg of sinapinic acid in 50 µl analyte solution (0.1% TFA dissolved in MilliQ H_2O). This mixture was further mixed in a 1:1 ratio with HPLC-grade acetonitrile to obtain the final matrix. The samples for MALDI TOF analysis were prepared in various ratios of protein:matrix, such as 1:1, 1:2, 1:5 and 1:10. The instrument comprises a MALDI ionization source, along with a TOF/TOF mass analyzer. Bruker Daltonics Flex Analysis software was used for obtaining and visualizing the spectra.

2.8. Preparation of DNA template for in vitro Transcription Experiments

The template DNA are synthesized by using an optimized multi-step overlapping PCR method[5] described by Suga *et al.*, for the generation of DNA templates of a flexizyme called eFx.[6] This DNA sequence contains a T7 promoter region, facilitating the binding of the T7 RNA polymerase towards the initiation of the T7 RNA polymerase-mediated transcription reaction. The primers used for this study have been depicted in **Table S3**.

Table S3: Sequences of primers used for the preparation of DNA for in vitro transcription experiments

Primer	Sequence
FxF	5'-GTAATACGACTCACTATAGGATCGAAAGATTTCCGC-3'
eFx-R1	5'-ACCTAACGCTAATCCCCTTTCGGGGCCGCGGAAATCTTTCGATCC-3'
T7-F	5'-GGCGTAATACGACTCACTATAG-3'
eFx-R2	5'-ACCTAACGCTAATCCCCT-3'

2.9. In vitro Transcription Reaction

The in vitro transcription reactions were setup following the protocol described by Suga *et al.*[7] The vials containing the transcription mixtures were incubated at 37°C for 4 hours followed by DNAse I treatment at the same temperature for an additional 30 minutes. The RNA from each vial was then extracted by standard phenol chloroform extraction method followed by ethanol precipitation. The purified RNA samples were then mixed with RNA loading buffer in a 1:1 ratio and heated at 95°C on a dry bath. They were then loaded on preheated 12% denaturing urea polyacrylamide gel and the gel electrophoresis took place for 1 hour at a constant current of 15mA. ssRNA ladder from New England Biolabs was used as the standard marker.[8,9] After

gel electrophoresis, the gel was taken out and washed with fresh 1X TBE buffer followed by staining of the gel with EtBr for 30 minutes. The gel was then visualized under a UV-transilluminator for analysing the RNA bands.[10]

2.10. In Vitro Transcription Kinetics Assay

The linear DNA templates containing a T7 promoter region prepared previously were then used for the kinetics studies. The DNA templates were extracted using a PCR clean-up kit (Macherey Nagel NucleoSpin Gel and PCR Clean-up kit), before conducting the transcription reactions. After purification, the concentration and purity of each template was quantified using a Nanodrop spectrophotometer (Implen NanoPhotometer N80) at a wavelength of 260 nm. Following this, the in vitro transcription reactions were set-up using either **^{SSB}T7 RNA Polymerase** (**^{TCNB}Ala$_{Ac}$** variant of T7 RNA Polymerase expressed via either an A1 or a A2 o-aaRS/tRNA pairs). The reactions were setup following a protocol described earlier.[11] The concentration of the enzyme was maintained at a constant concentration for all the experiments (final concentration = 0.1 mg/ml).

Each transcription reaction setup was incubated at 37°C and 20 µl aliquots were withdrawn at fixed time intervals of 0, 1, 2, 5, 10, 30, 60, 120 and 240 minutes respectively. The final amount of the DNA templates in each set of aliquots were set as 100 ng, 500 ng and 1000 ng respectively, for different sets of experiments. The transcription reaction of each withdrawn aliquot was quenched using a 1X stop buffer (8M urea, 50mM EDTA and tracking dye), followed by snap freezing on dry ice. All the experiments were performed in triplicates.

All the aliquots were then resolved on a denaturing urea-polyacrylamide gel following a process mentioned earlier. The band intensities corresponding to each RNA product were quantified using the densitometry technique on the ImageJ software. The values were then normalized against a standard ladder and the background was subtracted prior to plotting the kinetic curves. The band intensities were then plotted against time on the OriginPro software to generate the kinetic plots. The data were then fitted to a one-phase association model using nonlinear regression analysis in the same software:

$$Y = y_0 + A_1 . e^{\left(-x/t_1\right)}$$

Where,

Y = is the RNA yield at time t;
y_0 is the maximum product yield;
A_1 is the amplitude, and,
t_1 is the time constant of the initial transcription rate.

All the experiments were performed in triplicates and the standard errors in each plot were derived accordingly. Additionally, the R^2 values were analysed to assess the fitting quality of the curves. Finally, the comparison between the modified enzyme(s) and the wild type enzyme at individual DNA template concentrations was based on the both the yield (amplitude) and kinetic constant (t_1).

2.11. Molecular Docking Studies

2.11.A. Preparation of Macromolecule Structures for Docking

The **^{SSB}T7 RNA Polymerase** structure containing the **^{TCNB}Ala$_{Ac}$** residue at the 571st codon position was prepared using the PyMol software. The resulting PDB structure was then processed using the UCSF Chimera software (version 1.16). All the water molecules present in the PDB file were removed in order to avoid non-native clashes during molecular docking. The protein was then pre-processed using the software's inbuilt DockPrep module, which automatically added missing hydrogens and assigned atomic charges. After the structural clean-up, the protein was subjected to the energy minimization step using the software's "Minimize Structure" module. The minimization process involved 100 steps of steepest descent followed by 100 steps of conjugate gradient optimization, with a step size of 0.02Å for both the cases. None of the atoms were fixed during the energy minimization step, therefore allowing the complete relaxation of local torsions around the encoded FTUAA.

The eFx-DNA template containing a T7 promoter region was prepared with the help of the SCFBio IIT Delhi Nucleic Acid Builder (https://scfbio-iitd.res.in/software/drugdesign/bdna.jsp) online tool. The resulting double-stranded B-DNA was then checked for proper base-pairing, helical twist and finally the backbone geometry was analysed. The DNA model was then subjected to an energy minimisation process, following the same method as mentioned previously, before the molecular docking studies.

2.11.B. Docking Procedure

The **^{SSB}T7 RNA polymerase**-eFx template DNA docking was performed using the HDock server,[12–14] which is known to employ a hybrid template-based and ab initio algorithm specifically optimised for studying macromolecular interactions. The encoded T7 RNA Polymerase was designated as the input receptor molecule, whereas the eFx-DNA duplex was entered as the input ligand molecule. The molecular docking was performed under default parameters, and the top-ranked docked model was selected according to HDock's scoring function.[15]

Post docking, the docked model was analysed using PyMOL and Discovery Studio software. The positioning of the DNA, promoter alignment, domain orientation and possible steric interactions were analysed. Finally, the receptor-ligand interface residues were identified and compared with existing literature to validate our dataset. Additionally, a similar molecular docking study was performed using the WT-T7 RNAP and the eFx DNA template. A total of one hundred docked structures were generated by the HDock server for each protein sample, out of which the structure with the most favourable docking score was chosen for further studies.

3. Cloning and Site-Directed Mutagenesis

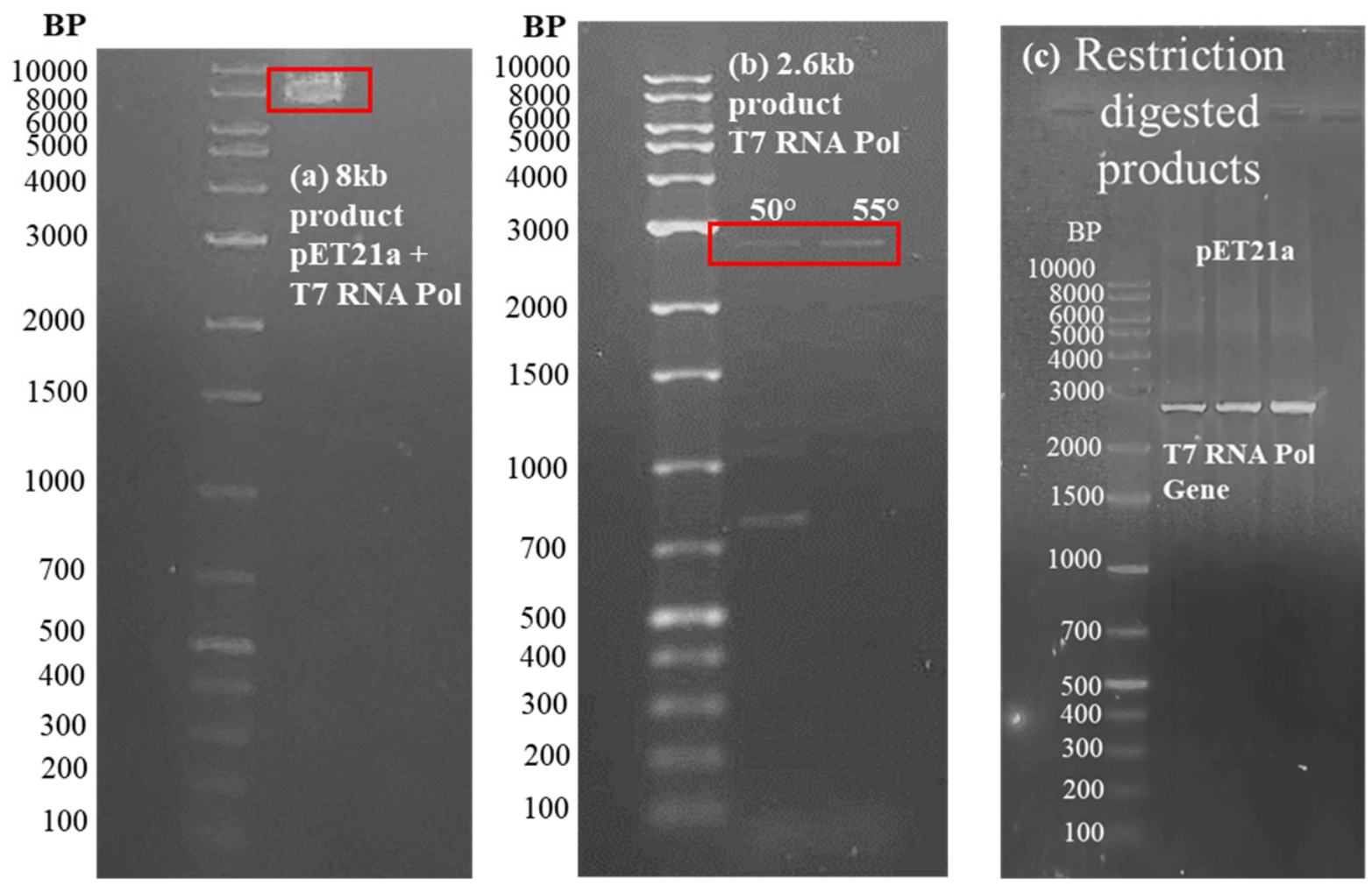

Figure S1. (a) Confirmation of the ligation of pET21a vector with the T7 RNA polymerase gene. The pET21a-T7 RNAP plasmid was isolated from a positive transformant and visualized on 0.7% agarose gel. A DNA band near 8kbp after digestion using a single restriction enzyme (BamHI) indicated the successful ligation of the vector and the gene. (b) Visualization of gradient PCR (Ta=50°C and 55°C) amplified T7 RNA polymerase gene using the pET21a-T7 RNAP plasmid as template. (c) Dual restriction enzyme (BamHI and EcoRI) digested T7 RNA polymerase gene and pET21a vector as visualized on EtBr stained 0.7% agarose gel.

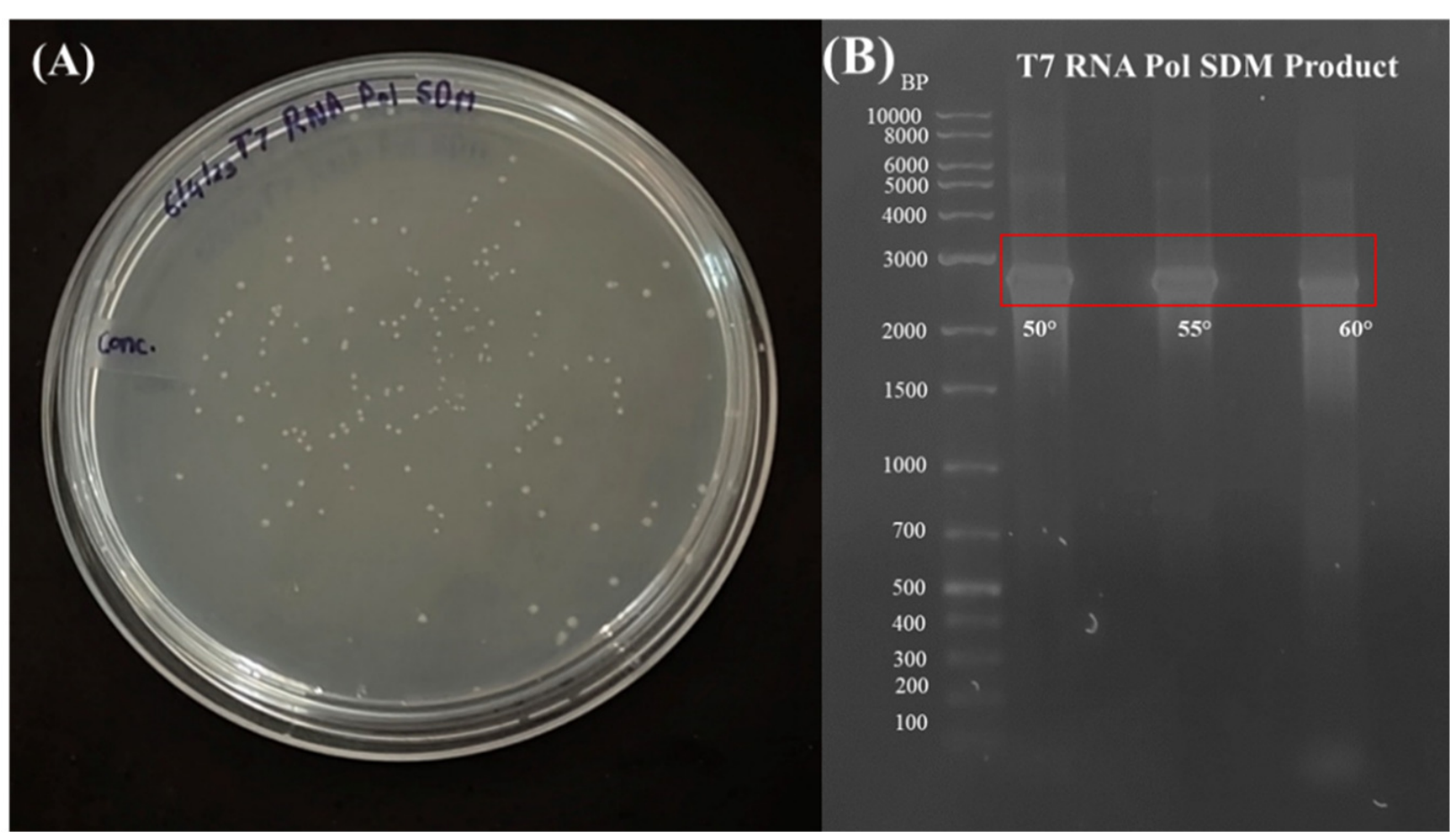

Figure S2. (A) Bacterial colonies formed on LB plate (Amp^R) after site-directed mutagenesis of the T7 RNA polymerase gene. (B) Confirmation of the formation of the T7 RNA Polymerase SDM product using gradient PCR reactions. The PCR amplified DNA bands near 2.6 kbp were visualized on EtBr stained 0.7% agarose gel indicating the formation of SDM products.

4. Plasmid Maps

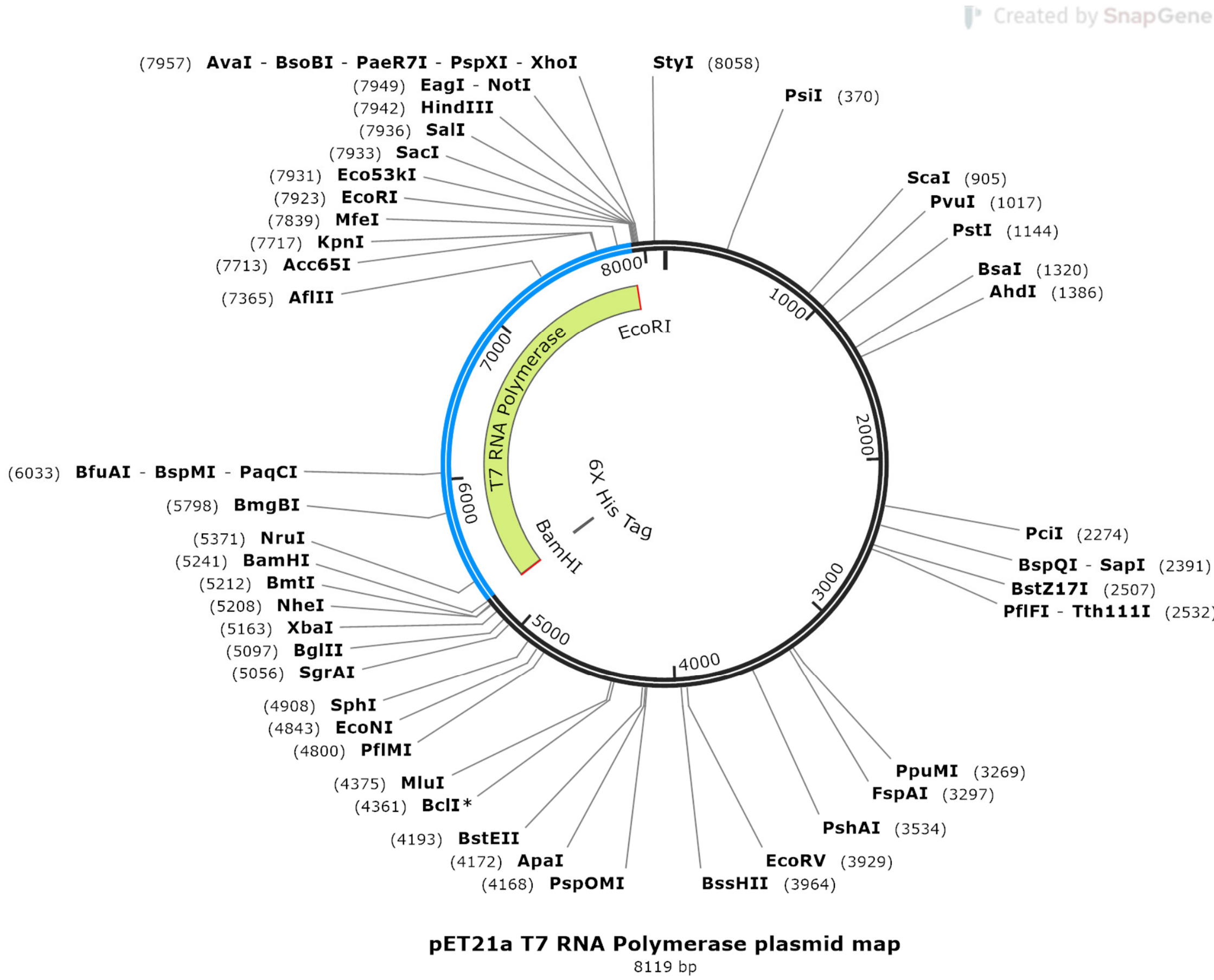

Figure S3. Plasmid map of T7 RNA Polymerase gene insert ligated to a pET21a vector (Amp^{R}).

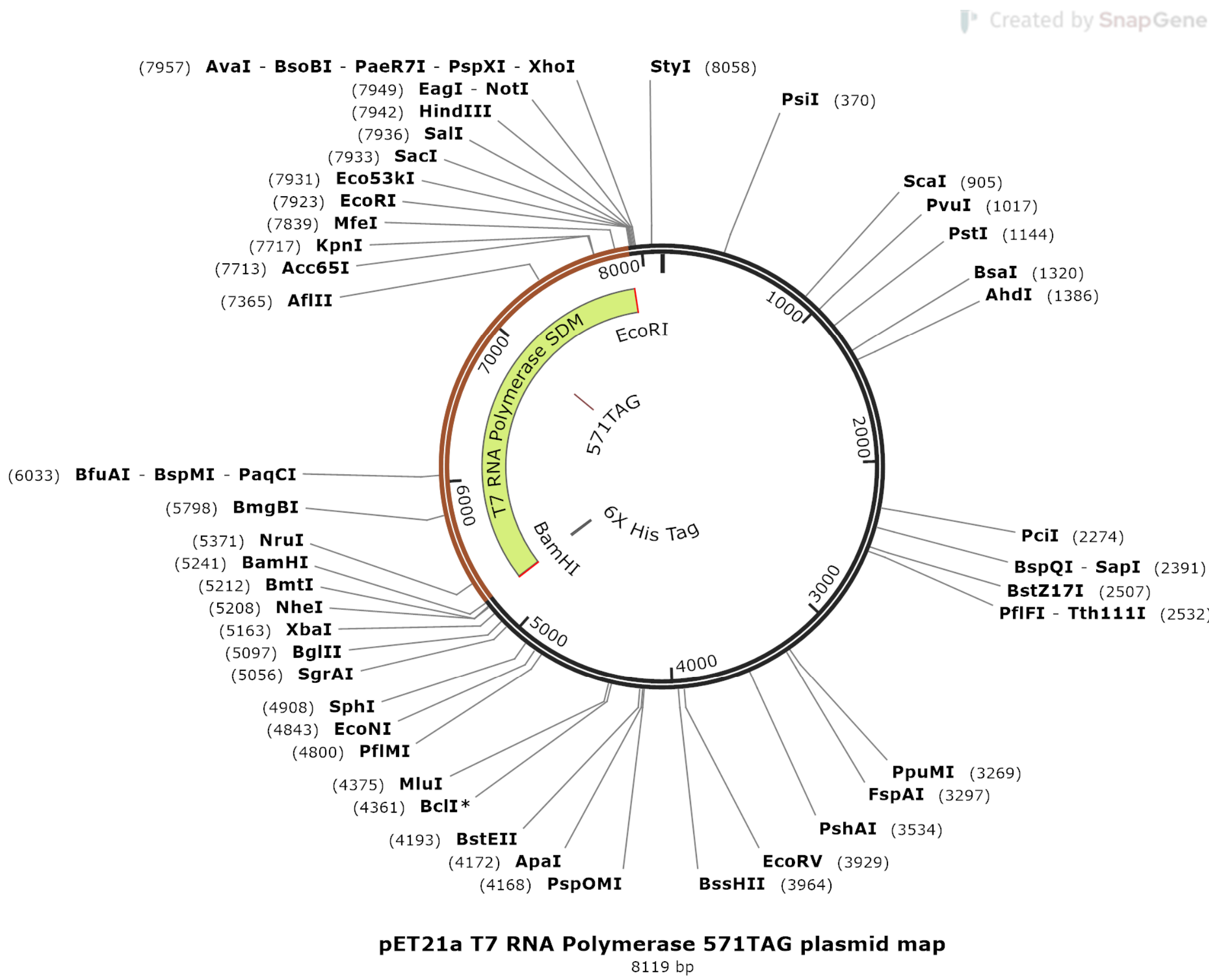

Figure S4. Plasmid map of T7 RNA Polymerase 571TAG gene insert ligated to a pET21a vector (Amp^R). The TAG stop codon at the 571st position has been additionally marked.

5. Protein Yield and SDS-PAGE Gel Electrophoresis Images

Table S4: Protein yield quantification of purified T7 RNAP variants (WT and FTUAA encoded) using Bradford assay

Sl No.	Protein Variant	Yield (mg/100 ml)	Yield % (of WT)
1	**WT-T7 RNAP**	3.5 ± 0.1	100 ± 2.8
2	**^{SSB}T7 RNAP (A1)**	2.9 ± 0.2	83 ± 5.7
3	**^{SSB}T7 RNAP (A2)**	3.1 ± 0.1	89 ± 2.8

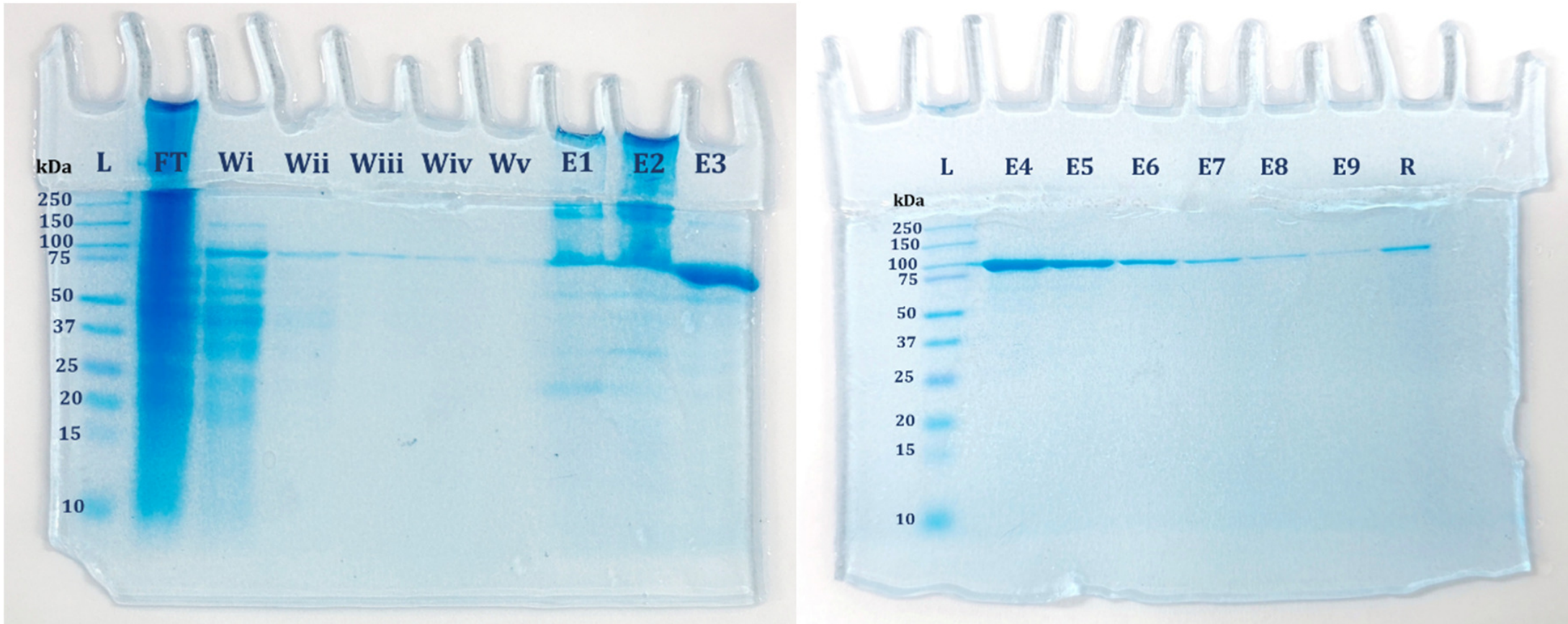

Figure S5. SDS-PAGE gel image depicting the formation of wild-type T7 RNA polymerase protein. Distinct protein bands (MW =~ 101 kDa) were observed close to the 100kDa molecular weight marker. The image also shows the presence of the protein bands in elution fractions 1-6, following which, the protein band intensity decreased multiple folds.

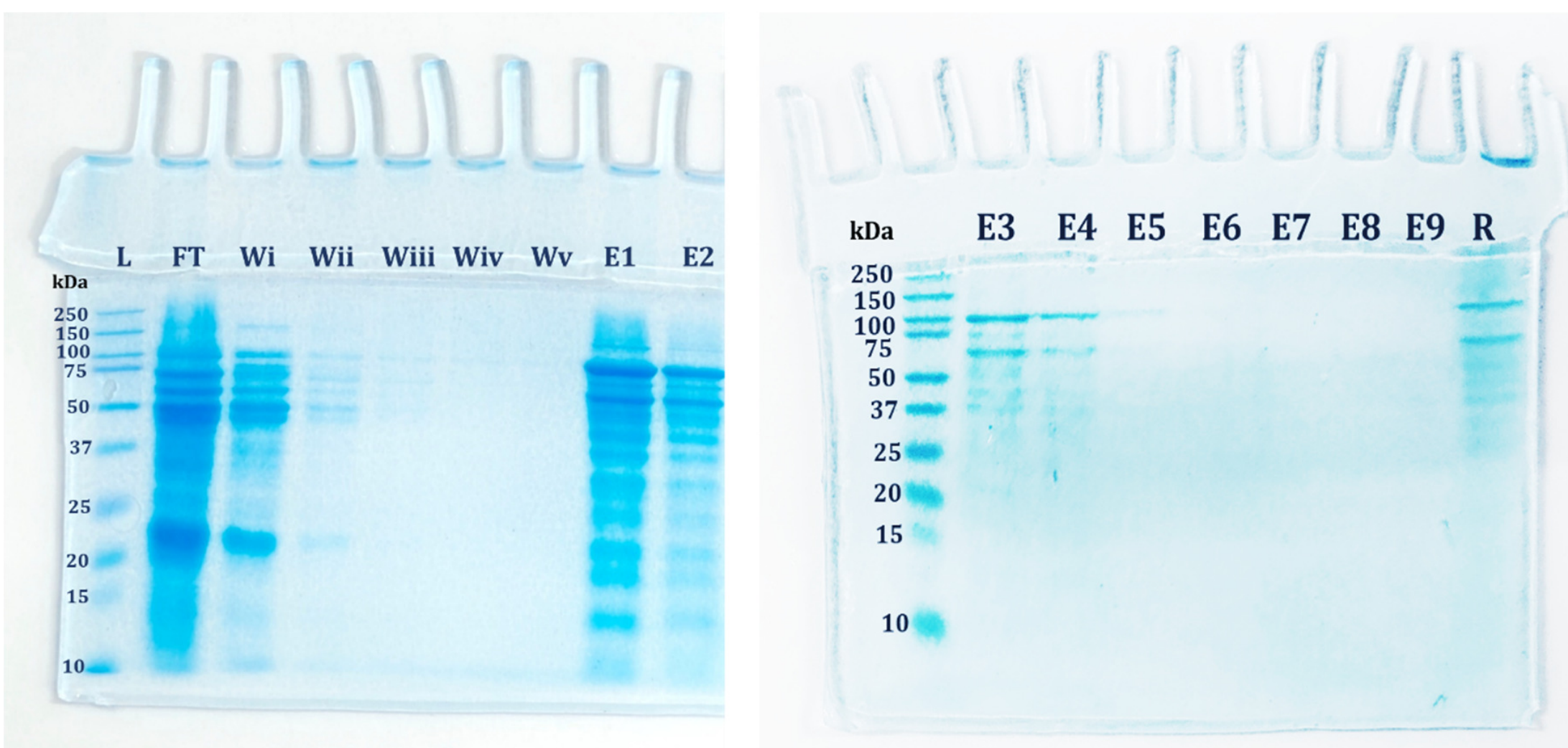

Figure S6. SDS-PAGE gel image depicting the formation of engineered T7 RNA polymerase containing $^{\mathbf{TCNB}}\mathbf{Ala}_{\mathbf{Ac}}$ at the 571st position, following the aminoacylation of the FTUAA by the A2 orthogonal aaRS/tRNA system. Distinct protein bands (MW =~ 101 kDa) were observed close to the 100kDa molecular weight marker. The image also shows the presence of the protein bands in elution fractions 1-4, following which, the protein band intensity decreased multiple folds.

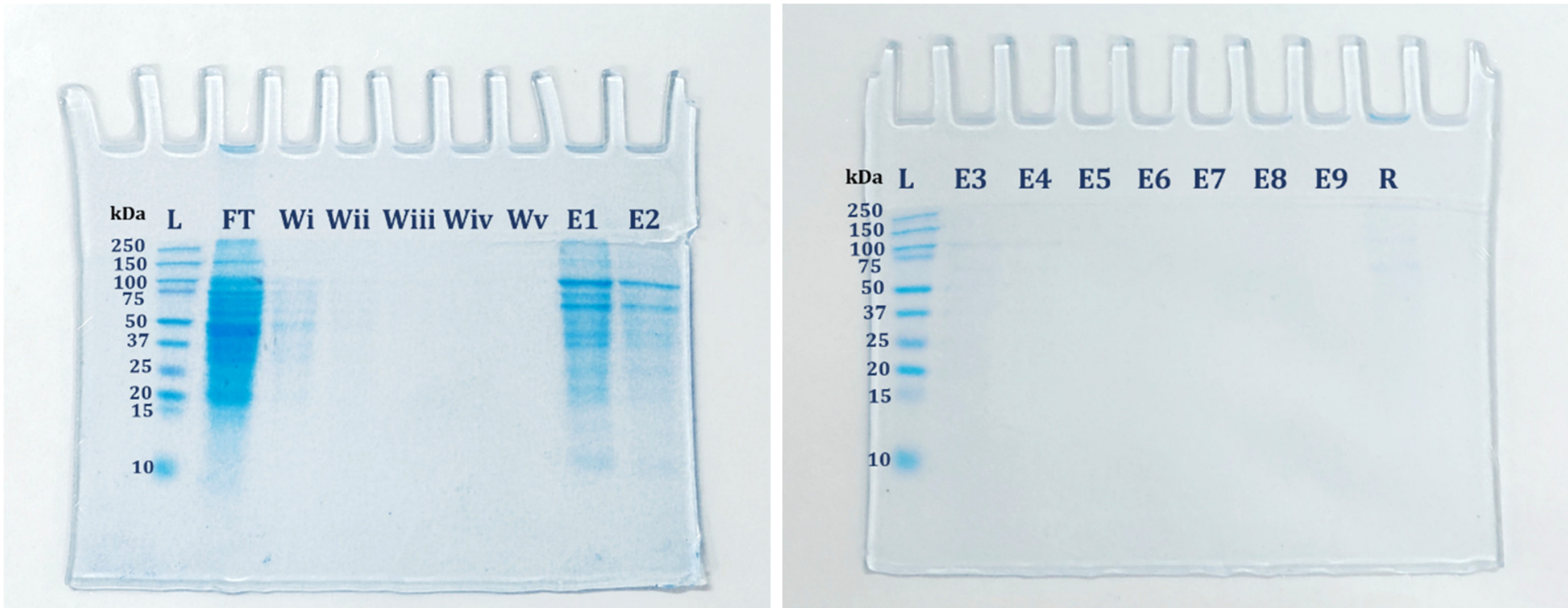

Figure S7. SDS-PAGE gel image depicting the formation of engineered T7 RNA polymerase containing $^{\mathbf{TCNB}}\mathbf{Ala_{Ac}}$ at the 571st position, following the aminoacylation of the FTUAA by the A1 orthogonal aaRS/tRNA system. Distinct protein bands (MW =~ 101 kDa) were observed close to the 100kDa molecular weight marker. The image also shows the presence of the protein bands in elution fractions 1-4, following which, the protein band intensity decreased multiple folds.

6. MALDI TOF Mass Spectrometry

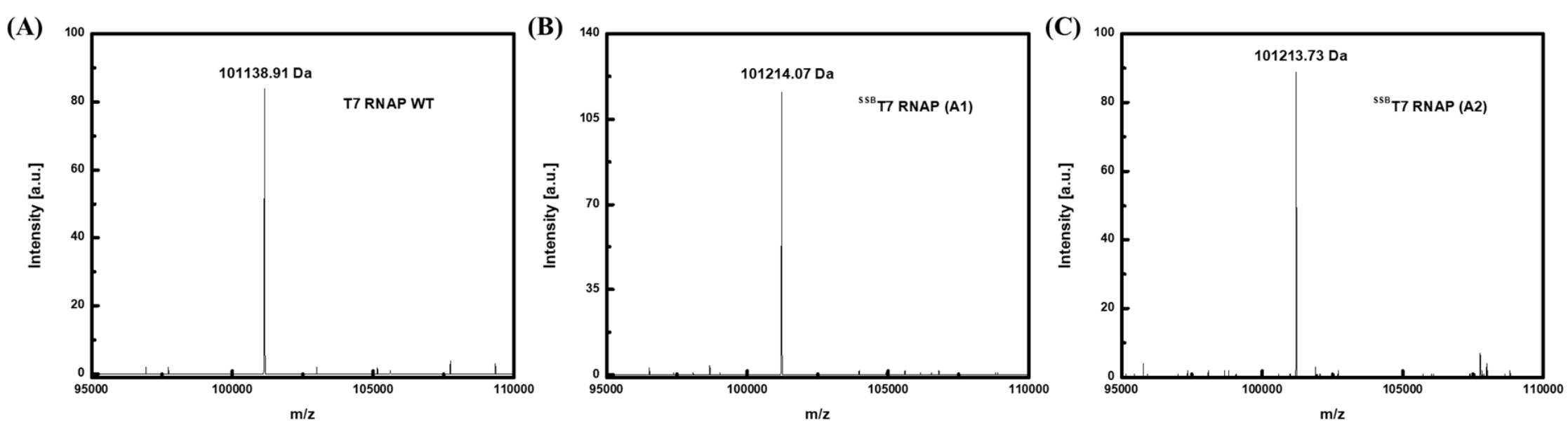

Figure S8. MALDI TOF spectra of (A) wild-type T7 RNA Polymerase, (B) ^{SSB}T7 RNA polymerase containing $^{\mathbf{TCNB}}\mathbf{Ala_{Ac}}$ incorporated by the A1 aaRS/tRNA pair and (C) ^{SSB}T7 RNA polymerase containing $^{\mathbf{TCNB}}\mathbf{Ala_{Ac}}$ incorporated by the A2 aaRS/tRNA pair.

Table S5: The mass shifts of the encoded ^{SSB}**T7 RNAPs** relative to the WT-T7 RNAP, confirming the successful incorporation of the FTUAAs.

Sl No	Protein Name	aaRS	Calculated Monosiotropic Molecular Weight (Da) (A)	Calculated ΔMass from WT (Da)	Molecular Weight (MALDI-TOF) (Da) (B)	Observed ΔMass from WT (Da)	Difference in Calculate and Experimental mass (A-B) (Da)
1	T7 RNAP WT	-	101138.29	NA	101138.91	NA	-0.62
2	T7 RNAP 571TAG	Tet3.0 (A1)	101214.31	+76.02 Da.	101214.07	+75.10	+0.24
3	T7 RNAP 571TAG	pylT-N346A-C348A (A2)	101214.31	+76.02 Da.	101213.73	+74.76	+0.58

7. Photophysical Studies

Table S6: Summary of the photophysical properties of the wild-type and FTUAA encoded ^{SSB}T7 RNA Polymerase proteins.

		UV-vis. and Fluorescence Properties				
**Entry**	**Solvent**	λ_{abs} **(nm)**	ε_{max}	λ_{Fl} **(nm)**	Φ_f	**Fluorescence Brightness**
Wild Type T7 RNA Polymerase	Dialysis Buffer	280 nm	ε^{280}_{max} = 94.06 * 10^3;	λ^{280}_{fl}= 332 nm;	0.096	9.03 * 10^3
		270 nm	ε^{270}_{max} = 95.07 * 10^3;	λ^{270}_{fl}= 333 nm;	0.099	9.41 * 10^3
		290 nm	ε^{290}_{max} = 77.88 * 10^3;	λ^{290}_{fl}= 333 nm;	0.076	5.92 * 10^3
^{SSB}T7 RNA Polymerase (A1)	Dialysis Buffer	280 nm	ε^{280}_{max} = 15.08 * 10^4;	λ^{280}_{fl}= 331 nm and 365 nm	0.55	8.30 * 10^4;
		270 nm	ε^{270}_{max} = 16.00 * 10^4;	λ^{270}_{fl}= 333 nm and 362 nm;	0.58	9.28 * 10^4;
		290 nm	ε^{290}_{max} = 78.95 * 10^3;	λ^{290}_{fl}= 320 nm and 370 nm;	0.40	3.16 * 10^4;
^{SSB}T7 RNA Polymerase (A2)	Dialysis Buffer	280 nm	ε^{280}_{max} = 15.08 * 10^4;	λ^{280}_{fl}= 331 nm and 365 nm;	0.53	7.99 * 10^4;

		270 nm	ε_{max}^{270} = 16.09 * 10^4;	λ_{fl}^{270}= 333 nm and 362 nm;	0.62	9.98 * 10^4;
		290 nm	ε_{max}^{290} = 78.95 * 10^3;	λ_{fl}^{290}= 320 nm and 370 nm;	0.43	3.40 * 10^4;

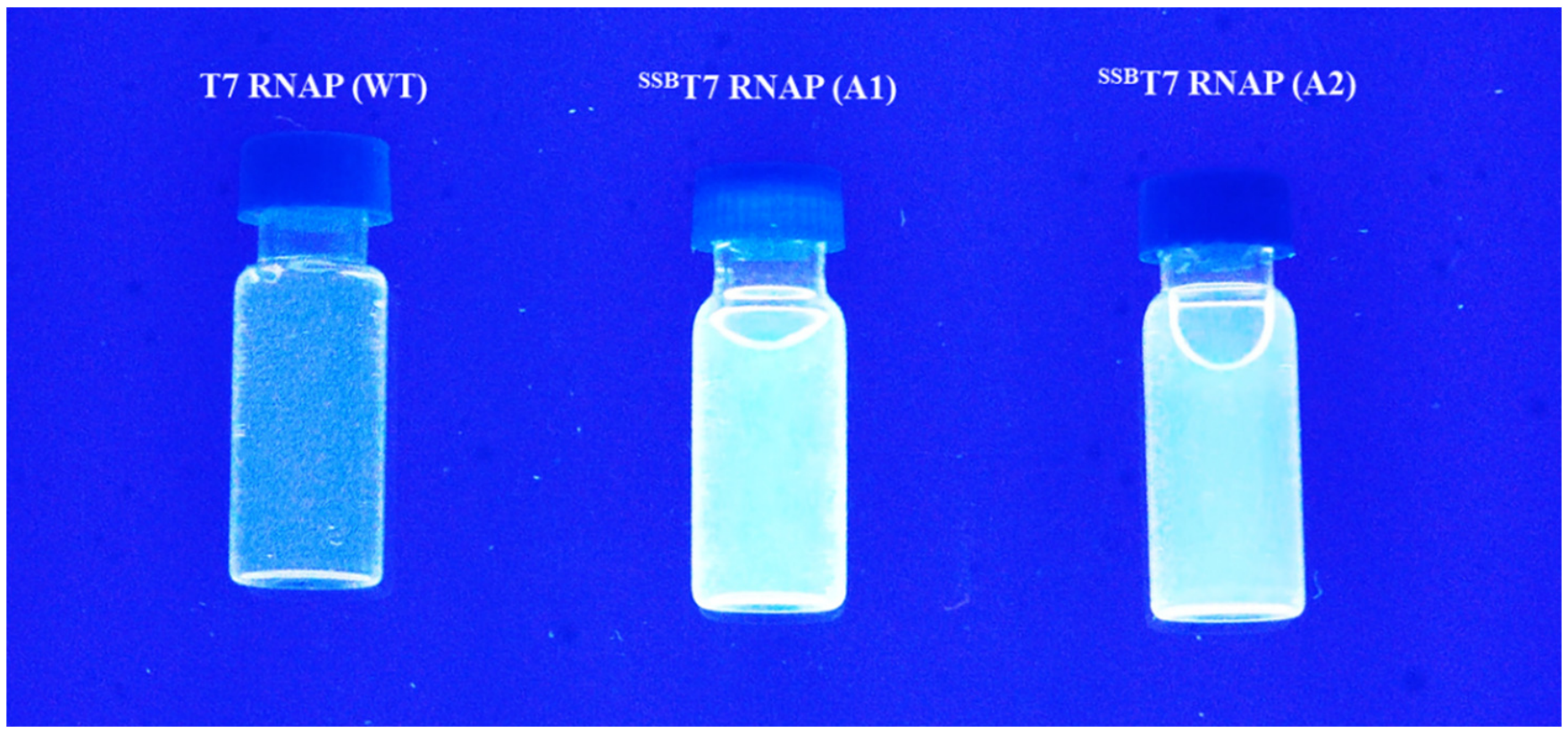

Figure S9. Visualization of the proteins under UV light (305 nm). From left to right: Wild type T7 RNA Polymerase; **SSBT7RNA Polymerase** containing the **TCNBAla$_{Ac}$** incorporated by the A1 aaRS system (Tet3.0) and **SSBT7RNA Polymerase** containing the **TCNBAla$_{Ac}$** incorporated by the A2 system (pylT-N346A-C348A).

8. In Vitro Transcription Studies

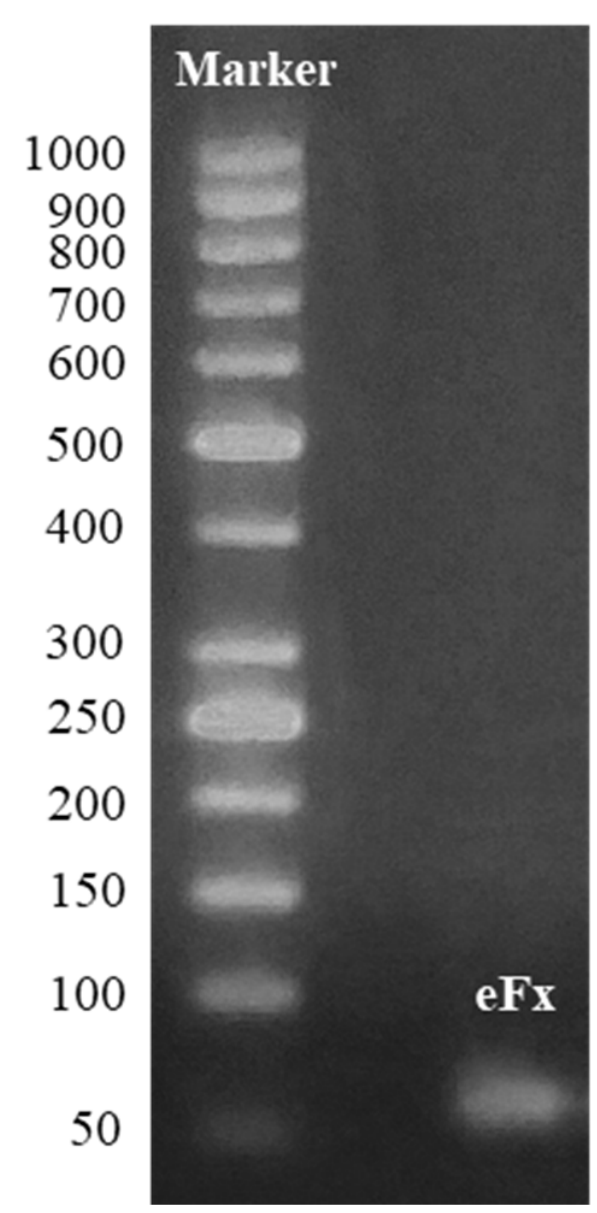

Figure S10. Visualization of the eFx DNA on EtBr stained 3% agarose gel.

9. Docking Poses and Various Interactions

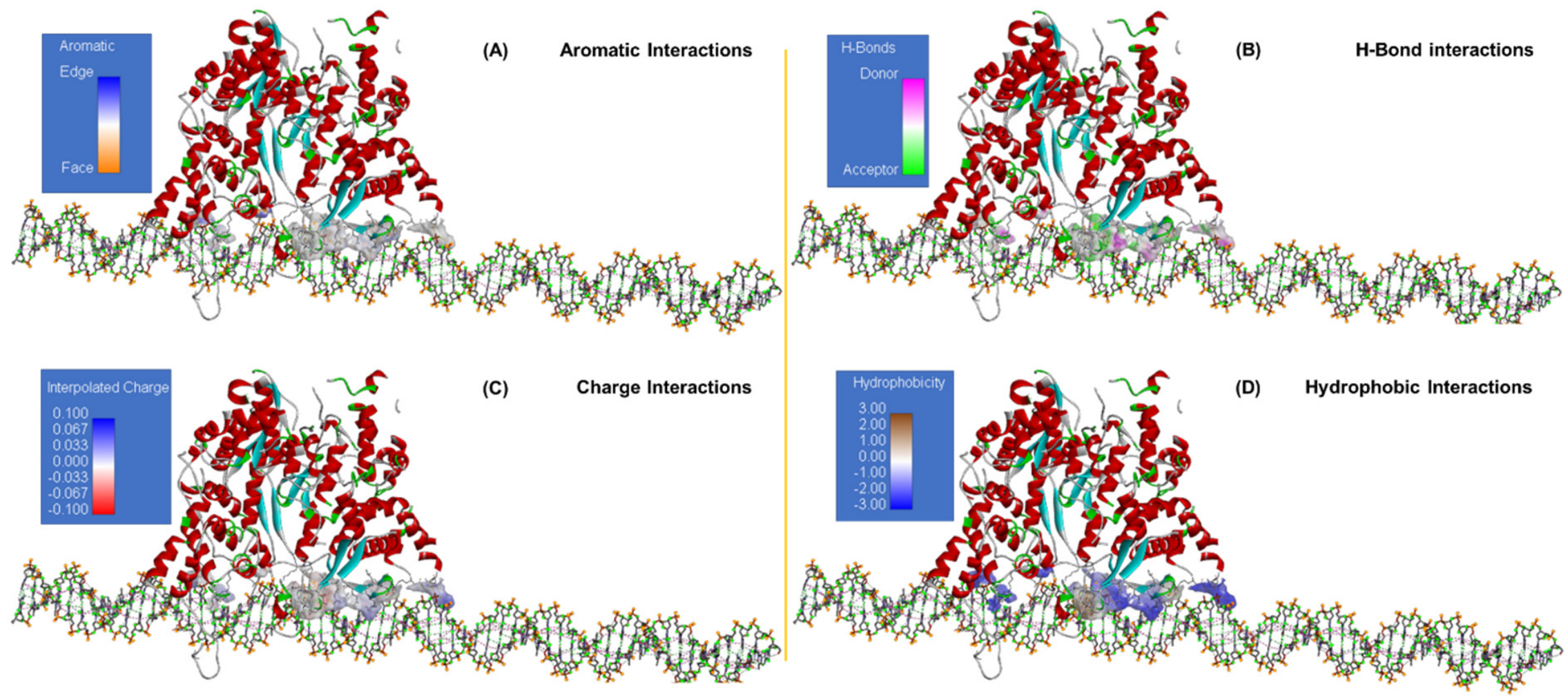

Figure S11. (A-D) Docked structures of the ^{SSB}**T7 RNA polymerase** bound to the eFx template DNA showing various interactions.

10. ^{1}H NMR Spectra of the Fluorescent Triazolyl Unnatural Amino Acid

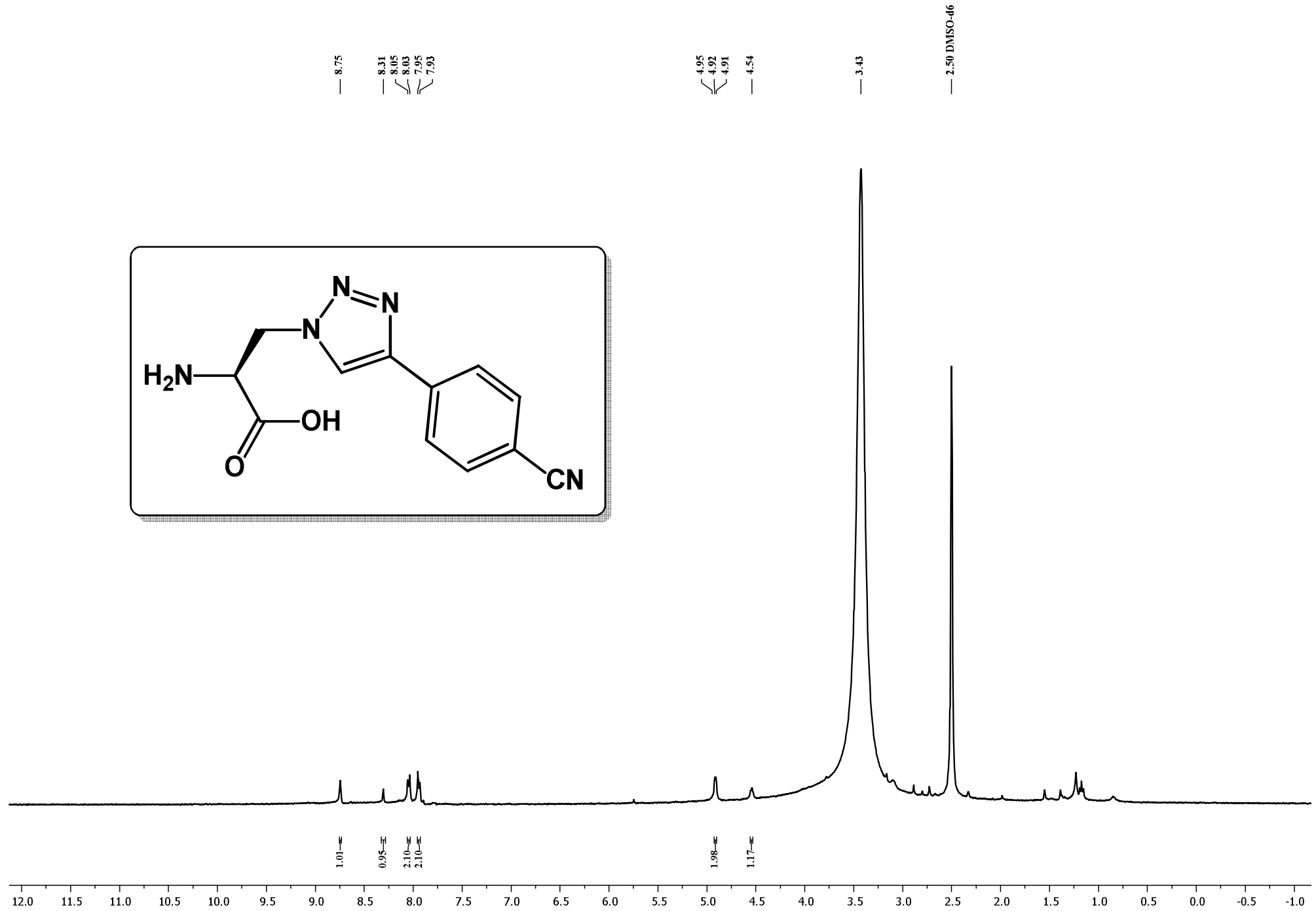

Figure S12. ^{1}H NMR spectra of $^{\mathbf{TCNB}}$**Ala$_{\mathbf{Ac}}$** (d_6-DMSO, 400MHz, 298K).

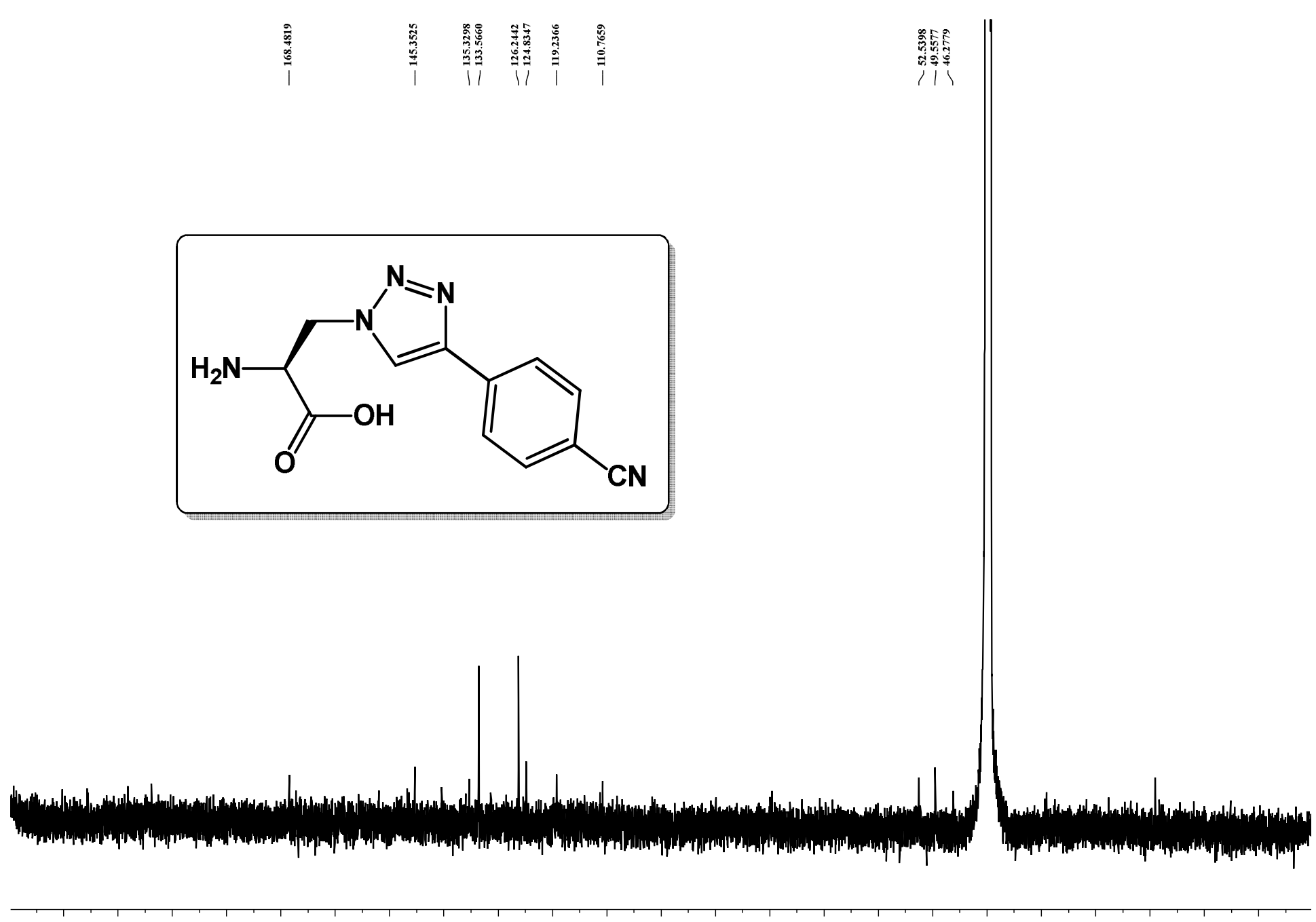

Figure S13. ^{13}C{^{1}H} NMR spectra of $^{\mathbf{TCNB}}$**Ala$_{\mathbf{Ac}}$** (d_6-DMSO, 500MHz, 298K).

11. References

(1) Wang, Y.-S.; Fang, X.; Wallace, A. L.; Wu, B.; Liu, W. R. A Rationally Designed Pyrrolysyl-TRNA Synthetase Mutant with a Broad Substrate Spectrum. *J. Am. Chem. Soc.* **2012**, *134* (6), 2950–2953.

(2) Jang, H. S.; Jana, S.; Blizzard, R. J.; Meeuwsen, J. C.; Mehl, R. A. Access to Faster Eukaryotic Cell Labeling with Encoded Tetrazine Amino Acids. *J. Am. Chem. Soc.* **2020**, *142* (16), 7245–7249.

(3) Yang, Z.; Chen, Z.; Zhang, Y. A Simple and Economical Site-Directed Mutagenesis Method for Large Plasmids by Direct Transformation of Two Overlapping PCR Fragments. *Biotechniques* **2022**, *73* (5), 227–233.

(4) Micsonai, A.; Wien, F.; Bulyáki, É.; Kun, J.; Moussong, É.; Lee, Y.-H.; Goto, Y.; Réfrégiers, M.; Kardos, J. BeStSel: A Web Server for Accurate Protein Secondary Structure Prediction and Fold Recognition from the Circular Dichroism Spectra. *Nucleic Acids Res.* **2018**, *46* (W1), W315–W322.

(5) MalagodaPathiranage, K.; Banerjee, R.; Martin, C. T. A New Approach to RNA Synthesis: Immobilization of Stably and Functionally Co-Tethered Promoter DNA and T7 RNA Polymerase. *Nucleic Acids Res.* **2024**, *52* (17), 10607–10618.

(6) Katoh, T.; Suga, H. Flexizyme-Catalyzed Synthesis of 3′-Aminoacyl-NH-TRNAs. *Nucleic Acids Res.* **2019**, *47* (9), e54–e54.

(7) Goto, Y.; Katoh, T.; Suga, H. Flexizymes for Genetic Code Reprogramming. *Nat. Protoc.* **2011**, *6* (6), 779–790.

(8) Oh, C. Y.; Henderson, E. R. In Vitro Transcription of Self-Assembling DNA Nanoparticles. *Sci. Rep.* **2023**, *13* (1), 12961.

(9) Wasinska-Kalwa, M.; Mamot, A.; Czubak, K.; Frankowska, K.; Rajkiewicz, A. A.; Spiewla, T.; Warminski, M.; Pilch, Z.; Szulc-Gasiorowska, M.; Siekan, K. Chemical Circularization of in Vitro Transcribed RNA for Exploring Circular MRNA Design. *Nat. Commun.* **2025**, *16* (1), 6455.

(10) Wang, Y.; Chen, Y.; Hu, Y.; Fang, X. Site-Specific Covalent Labeling of Large RNAs with Nanoparticles Empowered by Expanded Genetic Alphabet Transcription. *Proc. Natl. Acad. Sci.* **2020**, *117* (37), 22823–22832.

(11) Choi, Y.-K.; Katoh, T.; Beattie, A.; Suga, H. A Super Versatile Flexizyme System with Phenol Esters for Genetic Code Reprogramming. *Nat. Commun.* **2025**, *16* (1), 4671.

(12) Yan, Y.; Tao, H.; He, J.; Huang, S.-Y. The HDOCK Server for Integrated Protein–Protein Docking. *Nat. Protoc.* **2020**, *15* (5), 1829–1852.

(13) Yan, Y.; Zhang, D.; Zhou, P.; Li, B.; Huang, S.-Y. HDOCK: A Web Server for Protein–Protein and Protein–DNA/RNA Docking Based on a Hybrid Strategy. *Nucleic Acids Res.* **2017**, *45* (W1), W365–W373.

(14) Yan, Y.; Wen, Z.; Wang, X.; Huang, S. Addressing Recent Docking Challenges: A Hybrid Strategy to Integrate Template-based and Free Protein-protein Docking. *Proteins Struct. Funct. Bioinforma.* **2017**, *85* (3), 497–512.

(15) Huang, S.; Zou, X. An Iterative Knowledge-based Scoring Function for Protein–Protein Recognition. *Proteins Struct. Funct. Bioinforma.* **2008**, *72* (2), 557–579.
